# Association of collagen deep learning classifier with prognosis and chemotherapy benefits in stage II‐III colon cancer

**DOI:** 10.1002/btm2.10526

**Published:** 2023-04-17

**Authors:** Wei Jiang, Huaiming Wang, Weisheng Chen, Yandong Zhao, Botao Yan, Dexin Chen, Xiaoyu Dong, Jiaxin Cheng, Zexi Lin, Shuangmu Zhuo, Hui Wang, Jun Yan

**Affiliations:** ^1^ Department of General Surgery, Guangdong Provincial Key Laboratory of Precision Medicine for Gastrointestinal Tumor, Nanfang Hospital, The First School of Clinical Medicine Southern Medical University Guangzhou People's Republic of China; ^2^ School of Science Jimei University Xiamen Fujian People's Republic of China; ^3^ Department of Colorectal Surgery & Guangdong Institute of Gastroenterology, Guangdong Provincial Key Laboratory of Colorectal and Pelvic Floor Diseases, the Sixth Affiliated Hospital, Supported by National Key Clinical Discipline Sun Yat‐sen University Guangzhou Guangdong People's Republic of China; ^4^ Department of Pathology, the Sixth Affiliated Hospital Sun Yat‐sen University Guangzhou Guangdong People's Republic of China

**Keywords:** chemotherapy benefits, Collagen^DL^ classifier, colon cancer, deep learning, prognosis

## Abstract

The current tumor‐node‐metastasis staging system does not provide sufficient prognostic prediction or adjuvant chemotherapy benefit information for stage II‐III colon cancer (CC) patients. Collagen in the tumor microenvironment affects the biological behaviors and chemotherapy response of cancer cells. Hence, in this study, we proposed a collagen deep learning (collagen^DL^) classifier based on the 50‐layer residual network model for predicting disease‐free survival (DFS) and overall survival (OS). The collagen^DL^ classifier was significantly associated with DFS and OS (*P* < 0.001). The collagen^DL^ nomogram, integrating the collagen^DL^ classifier and three clinicopathologic predictors, improved the prediction performance, which showed satisfactory discrimination and calibration. These results were independently validated in the internal and external validation cohorts. In addition, high‐risk stage II and III CC patients with high‐collagen^DL^ classifier, rather than low‐collagen^DL^ classifier, exhibited a favorable response to adjuvant chemotherapy. In conclusion, the collagen^DL^ classifier could predict prognosis and adjuvant chemotherapy benefits in stage II‐III CC patients.

## 1 INTRODUCTION

Colon cancer (CC) is one of the main causes of cancer mortality worldwide.^1^ At present, patients with CC are treated based on the tumor‐node‐metastasis (TNM) staging system, which has been widely used in the clinic and has been continuously revised to the eighth edition.^2^ Nevertheless, the TNM staging system cannot adequately distinguish the prognosis of stage II‐III CC patients, especially those who receive adjuvant chemotherapy.^3^, ^4^ The 5‐year overall survival (OS) of these patients is between 50% and 90%, which indicates that the TNM system cannot provide sufficient prognosis and adjuvant chemotherapy benefit information. Therefore, there is an urgent need for an efficient biomarker to complement the current TNM staging system to improve the accuracy of treatment selection and prognosis prediction.

The scaffolding of the tumor microenvironment (TME) is composed of the extracellular matrix (ECM), which could impact the biological behavior of cancer cells.^5^, ^6^ Collagen is the main component of the ECM and plays a major role in ECM function.^7^, ^8^ Some studies have proven that collagen in the TME is a valuable marker for evaluating the prognostic outcomes of patients with gastrointestinal tumors.^9^, ^10^, ^11^ In addition, the stiffness of tumor tissue could increase because of abnormal collagen cross‐linking and deposition, which leads to chemotherapy resistance.^6^, ^8^, ^12^ Thus, we hypothesize that collagen in the TME is an effective biomarker to provide prognosis and chemotherapy benefit information in stage II‐III CC patients.

Multiphoton imaging, based on nonlinear optics and femtosecond lasers, can acquire the collagen structure and cell morphology of biological samples. The second harmonic generation (SHG) signal is produced from collagen fiber, and the two‐photon excitation fluorescence is produced by cells.^13^, ^14^ Due to its imaging principle and physical origin, multiphoton imaging has high selectivity and specificity for collagen. Therefore, multiphoton imaging can accurately capture information about the conformational alterations of collagen in the TME.

Currently, several machine‐learning algorithms have been applied to explore disease information in medical images. Among them, deep learning (DL) is one of the most effective approaches used to perform medical image recognition and classification.^15^, ^16^, ^17^, ^18^, ^19^ Moreover, DL has promising potential to predict prognosis by extracting prognosis‐associated information from medical images.^20^, ^21^ Hence, this study aimed to construct a collagen‐deep‐learning (collagen^DL^) prognostic classifier based on multiphoton imaging and DL for effectively predicting the survival of patients with stage II‐III CC and explore whether the collagen^DL^ classifier could identify patients with high‐risk stage II and III CC who might benefit from adjuvant chemotherapy.

## 2 RESULTS

### 2.1 Patient characteristics

The clinicopathologic characteristics of the patients in the training, internal validation, and external validation cohorts are shown in Table 1. The distribution of clinicopathological characteristics was similar among the three cohorts. A total of 882 patients were included in this study: 299 (33.9%) patients were older than 65 years, 491 (55.7%) patients were male, and 495 (56.1%) patients received adjuvant chemotherapy. The median disease‐free survival (DFS) times were 60, 59, and 48 months, and the median OS times were 61, 60, and 52 months in the three cohorts, respectively.

**TABLE 1:** Characteristics of the patients in the training and validation cohorts.

Characteristic	Training cohort (*n* = 303)	Internal validation cohort (*n* = 287)	External validation cohort (*n* = 292)	*P* value
Age, years old, No. (%)				
<65	199 (65.7)	195 (67.9)	189 (64.7)	0.706
≥65	104 (34.3)	92 (32.1)	103 (35.3)	
Sex, No. (%)				
Male	185 (61.1)	155 (54.0)	151 (51.7)	0.057
Female	118 (38.9)	132 (46.0)	141 (48.3)	
Bowel obstruction, No. (%)				
No	231 (76.2)	221 (77.0)	194 (66.4)	0.006
Yes	72 (23.8)	66 (23.0)	98 (33.6)	
Primary tumor location, No. (%)				
Left sided	182 (60.1)	169 (58.9)	178 (61.0)	0.878
Right sided	121 (39.9)	118 (41.1)	114 (39.0)	
Preoperative CEA level, No. (%)				
Normal	181 (59.7)	178 (62.0)	185 (63.4)	0.655
Elevated	122 (40.3)	109 (38.0)	107 (36.6)	
Tumor budding, No. (%)				
Low or intermediate	225 (74.3)	204 (71.1)	213 (72.9)	0.685
High	78 (25.7)	83 (28.9)	79 (27.1)	
Tumor differentiation, No. (%)				
Well or moderately	235 (77.6)	227 (79.1)	223 (76.4)	0.733
Poorly or undifferentiated	68 (22.4)	60 (20.9)	69 (23.6)	
VELIPI, No. (%)				
No	123 (40.6)	119 (41.5)	133 (45.5)	0.430
Yes	180 (59.4)	168 (58.5)	159 (54.5)	
Tumor size, cm, No. (%)				
<4	132 (43.6)	121 (42.2)	121 (41.4)	0.867
≥4	171 (56.4)	166 (57.8)	171 (48.6)	
T stage, No. (%)				
T1–T3	152 (50.2)	154 (53.7)	153 (52.4)	0.690
T4	151 (49.8)	133 (46.3)	139 (47.6)	
N stage, No. (%)				
N0	161 (53.1)	148 (51.6)	155 (53.1)	0.384
N1	85 (28.1)	75 (26.1)	90 (30.8)	
N2	57 (18.8)	64 (22.3)	47 (16.1)	
TNM stage, No. (%)				
II	161 (53.1)	148 (51.6)	155 (53.1)	0.912
III	142 (46.9)	139 (48.4)	137 (46.9)	
Adjuvant chemotherapy, No. (%)				
No	138 (45.5)	131 (45.6)	118 (41.4)	0.345
Yes	165 (54.5)	156 (54.4)	174 (58.6)	
Collagen^DL^ classifier, No. (%)				
Low	147 (48.5)	136 (47.4)	149 (51.0)	0.668
High	156 (51.5)	151 (52.6)	143 (49.0)	

*Note*: Values in parentheses are percentages unless indicated otherwise.

Abbreviations: CEA, carcinoembryonic antigen; TNM, tumor‐node‐metastasis; VELIPI, venous emboli and/or lymphatic invasion and/or perineural invasion.

### 2.2 Collagen^DL^ classifier and prognosis

**FIGURE 1:**
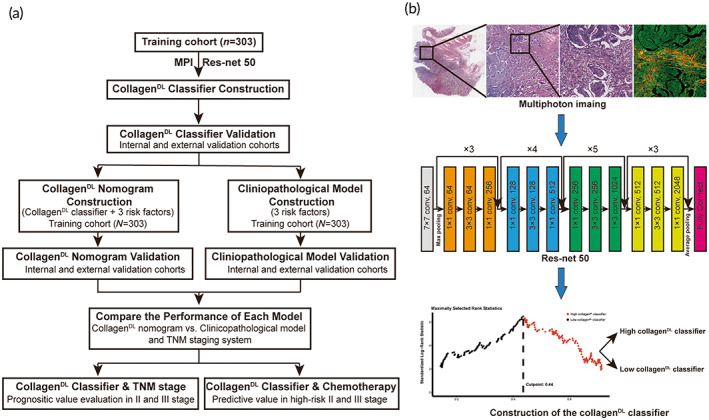
The workflow of this study. (a) Study design of this study. (b) Flow chart of the collagen^DL^ classifier. A representative ROI with a field of view of 512 × 512 μm was chosen in the HE staining image, and the corresponding multiphoton image was obtained. Then, the probability value of recurrence from these multiphoton images is output through Res‐net 50. Finally, the collagen^DL^ classifier was constructed according to the optimal cut‐off probability value. HE, hematoxylin and eosin; MPI, multiphoton imaging; Res‐net 50, 50‐layer residual network; ROI, region of interest.

**FIGURE 2:**
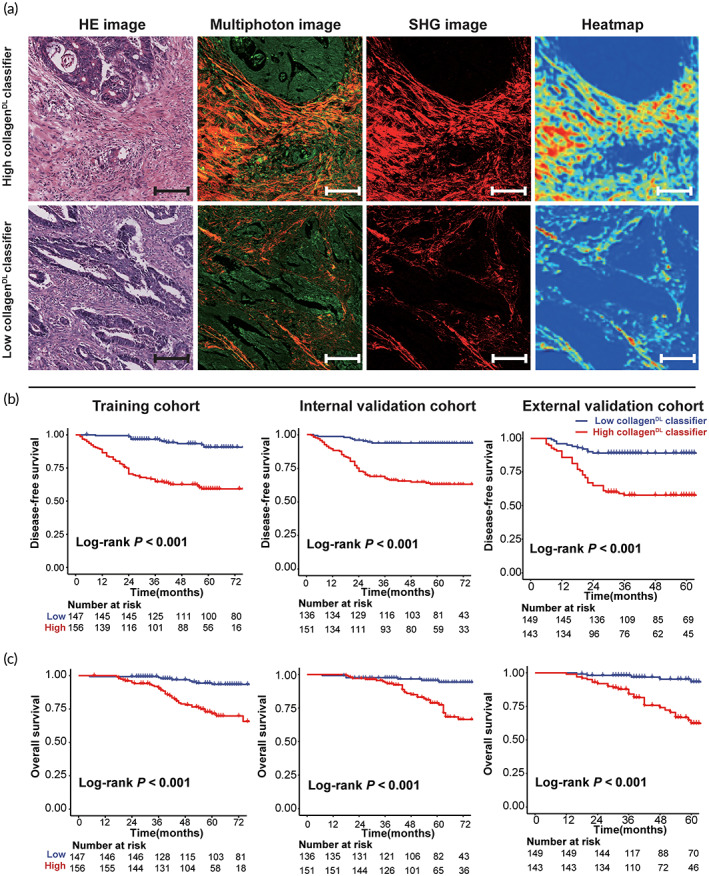
Representative collagen^DL^ classifier in patients with different prognoses. (a) Representative HE image, corresponding multiphoton image (collagen is illustrated in red), SHG image, and heatmap of patients with high‐ and low‐collagen^DL^ classifier. The color gradient of the heatmap corresponds to the contribution of a pixel towards recurrence in patients with stage II‐III CC, with darker colors showing a greater contribution. Survival curves of DFS (b) and OS (c) for patients with high‐ or low‐ collagen^DL^ classifier in the three cohorts. Scale bars: 100 μm. CC, colon cancer; DFS, disease‐free survival; HE, hematoxylin and eosin; OS, overall survival; SHG, second harmonic generation.

The workflow of this study is shown in Figure 1. All patients were divided into high‐ and low‐collagen^DL^ classifier subgroups according to the optimal cut‐off recurrence probability value (0.436) in the training cohort (Figure S1). The relationships between clinicopathological characteristics and the collagen^DL^ classifier are shown in Table S1. Representative images of the high‐ and low‐collagen^DL^ classifier subgroups are shown in Figure 2a. In the training cohort, the 5‐year DFS and OS rates were 90.6% and 93.2% for patients with a low collagen^DL^ classifier and 59.0% and 68.6% for patients with a high collagen^DL^ classifier, respectively (*P* < 0.001). Then, the collagen^DL^ classifier was applied to the internal and external validation cohorts, and the results demonstrated that the collagen^DL^ classifier could also significantly distinguish patients with different prognoses in these cohorts (Figure **2**b,c).

In the training cohort, the collagen^DL^ classifier yielded Harrell's concordance indexes (C‐indexes) of 0.699 for DFS and 0.678 for OS. Good discrimination was also verified in the internal and external validation cohorts, with C‐indexes of 0.692 and 0.697 for DFS and 0.660 and 0.692 for OS, respectively. In the three cohorts, the 5‐year time‐dependent receiver operating characteristic (ROC) curves also confirmed that the collagen^DL^ classifier has good discrimination in terms of predicting prognosis (Figure S2).

**FIGURE 3:**
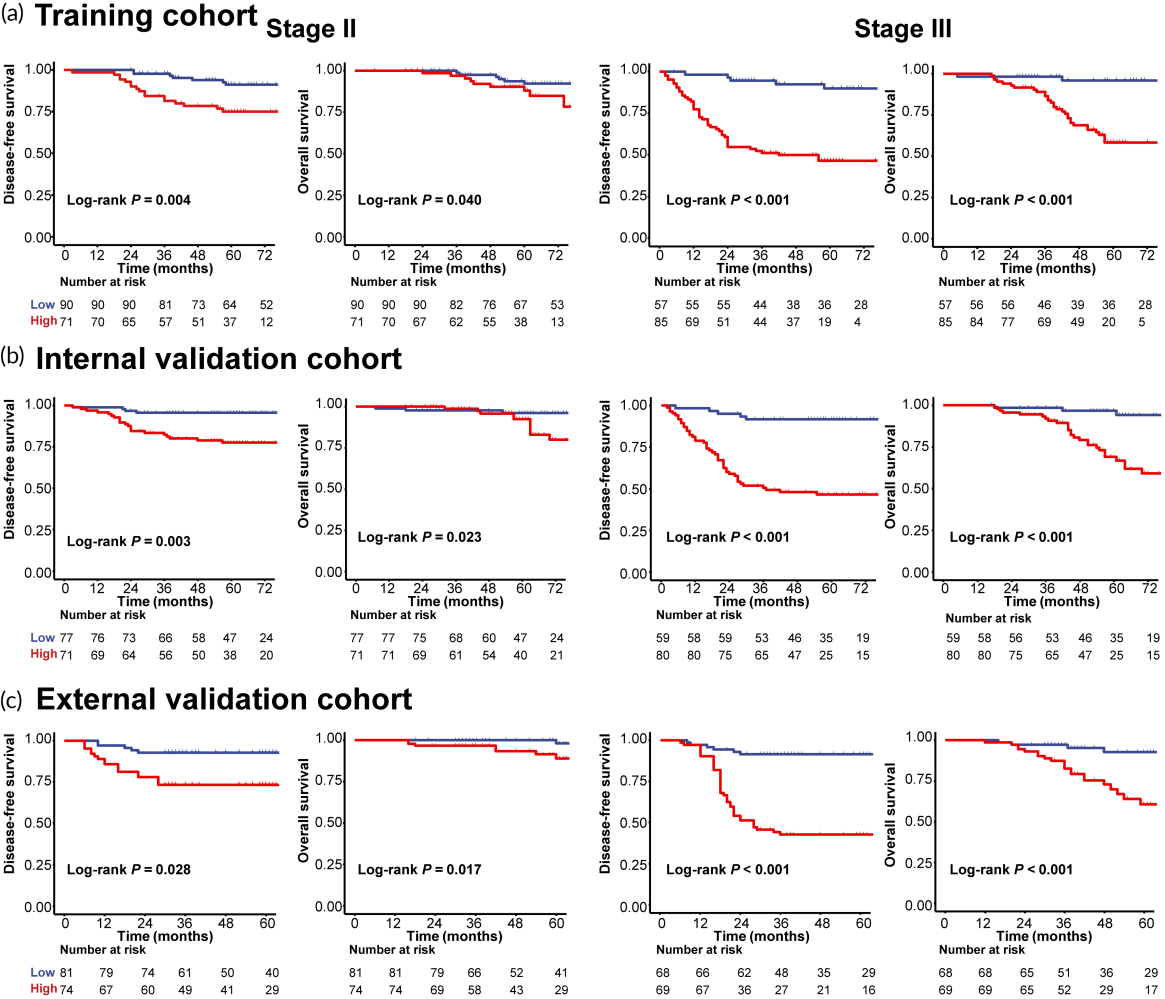
Kaplan–Meier survival analyses of **DFS** and **OS** based on the **collagen** ^**DL**^ classifier in subgroups of **CC** patients in the three cohorts. (a) Training cohort. (b) Internal validation cohort. (c) External validation cohort. *P* values were calculated by the log‐rank test.

Stratified analyses of patients with stage II and III CC indicated that the collagen^DL^ classifier was a valuable biomarker to identify stage II‐III patients with different prognoses in the three cohorts (Figure 3). When each clinicopathological characteristic was used for stratified analysis, the collagen^DL^ classifier could still distinguish patients with different DFS and OS rates (Figure S3–S8).

### 2.3 Individualized collagen^DL^ nomogram construction and performance assessment

**FIGURE 4:**
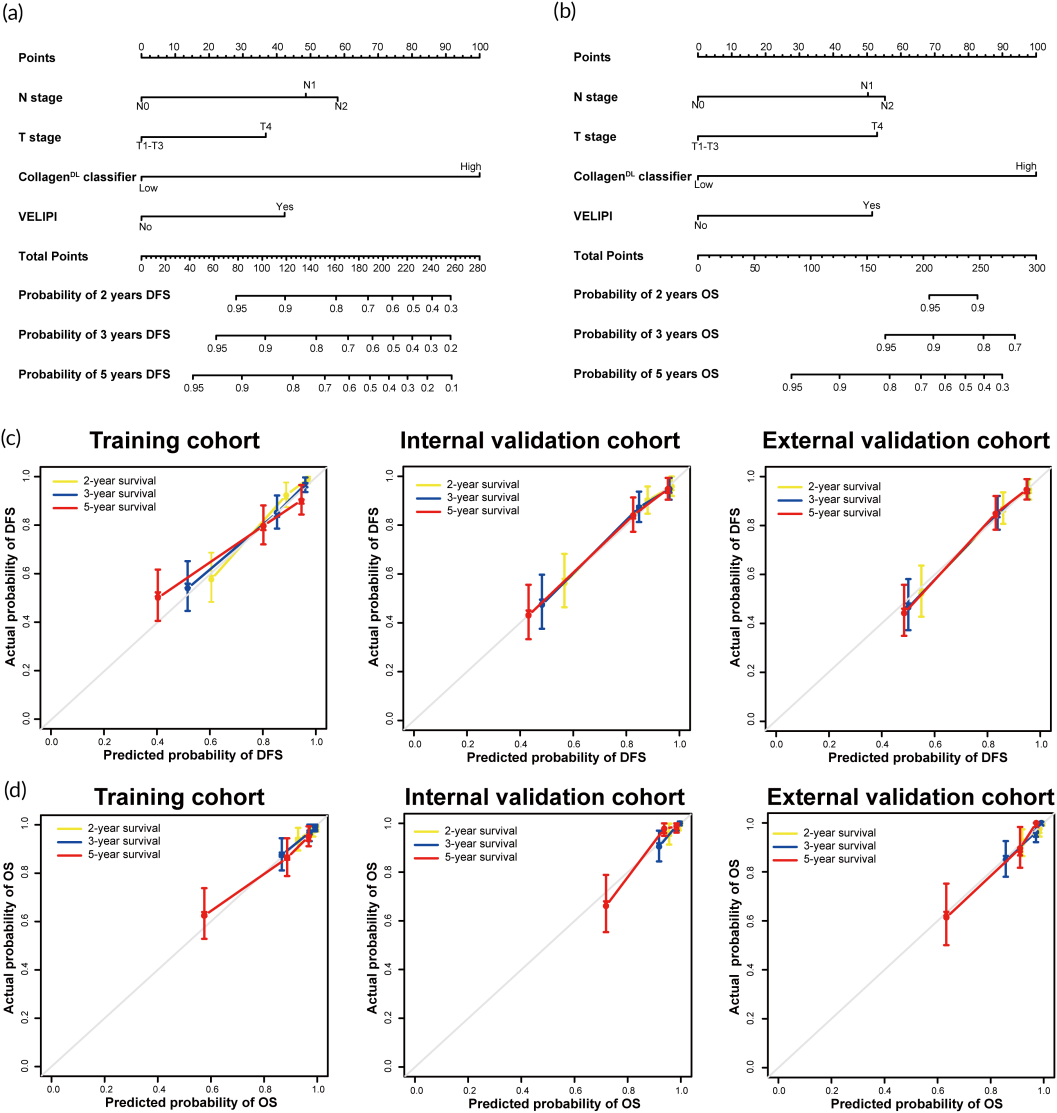
Development of the **collagen** ^**DL**^ nomograms to estimate **DFS** and **OS** for **CC** patients with stage **II‐III** disease. (a) Individualized collagen^DL^ nomogram for DFS. (b) Individualized collagen^DL^ nomogram for OS. The collagen^DL^ nomograms were construed by integrating T stage, N stage, VELIPI, and the collagen^DL^ classifier. Calibration curves of the collagen^DL^ nomograms for DFS (c) and OS (d) in the training, internal validation, and external validation cohorts. Calibration curves show the calibration of the collagen^DL^ nomograms in terms of the agreement between the predicted and actual 2‐, 3‐, and 5‐year outcomes. DFS, disease‐free survival; OS, overall survival; VELIPI, venous emboli and/or lymphatic invasion and/or perineural invasion.

Univariate Cox regression analysis demonstrated that venous emboli and/or lymphatic invasion and/or perineural invasion (VELIPI), T stage, N stage, and the collagen^DL^ classifier were candidate predictors of prognosis in the training cohort (Table S2). Multivariable Cox regression analyses indicated that the collagen^DL^ classifier was an independent predictor of DFS and OS after adjustment for clinicopathological characteristics in the training cohort (hazard ratio [HR]: 5.848 [95% CI: 3.140–10.890; *P* < 0.001] and 5.382 [95% CI: 2.538–11.411; *P* < 0.001]) (Table 2). According to the results of multivariable Cox regression analyses, two individualized nomograms for predicting DFS and OS integrating the collagen^DL^ classifier, VELIPI, T stage, and N stage were developed (Figure 4a,b). The collagen^DL^ nomograms demonstrated satisfactory discrimination and calibration in the three cohorts with C‐indexes of 0.788 for DFS and 0.777 for OS in the training cohort, 0.795 for DFS and 0.763 for OS in the internal validation cohort, and 0.778 for DFS and 0.822 for OS in the external validation cohort. The calibration curves of the two collagen^DL^ nomograms displayed good agreement between the collagen^DL^ nomogram‐predicted survival and actual survival at 2, 3, and 5 years (Figure 4c,d).

**TABLE 2:** Multivariable Cox regression analyses for disease‐free and overall survival in the training cohort.

Characteristic	Disease‐free survival		Overall survival	
	HR (95% CI)	*P*	HR (95% CI)	*P*
VELIPI				
No	1 [Reference]		1 [Reference]	
Yes	2.108 (1.244, 3.571)	0.006	2.375 (1.226, 4.603)	0.010
T stage				
T1–T3	1 [Reference]		1 [Reference]	
T4	1.909 (1.187, 3.068)	0.008	2.434 (1.331, 4.450)	0.008
N stage				
N0	1 [Reference]		1 [Reference]	
N1	2.356 (1.342, 4.139)	0.003	2.323 (1.157, 4.665)	0.018
N2	2.761 (1.557, 4.895)	0.001	2.529 (1.274, 5.021)	0.008
Collagen^DL^ classifier				
Low	1 [Reference]		1 [Reference]	
High	5.848 (3.140, 10.890)	<0.001	5.382 (2.538, 11.411)	<0.001

Abbreviations: CI, confidence interval; CEA, carcinoembryonic antigen; HR, hazard ratio; NA, not available; VELIPI, venous emboli and/or lymphatic invasion and/or perineural invasion.

### 2.4 Assessment of the incremental value of the collagen^DL^ classifier in predicting DFS and OS

**FIGURE 5:**
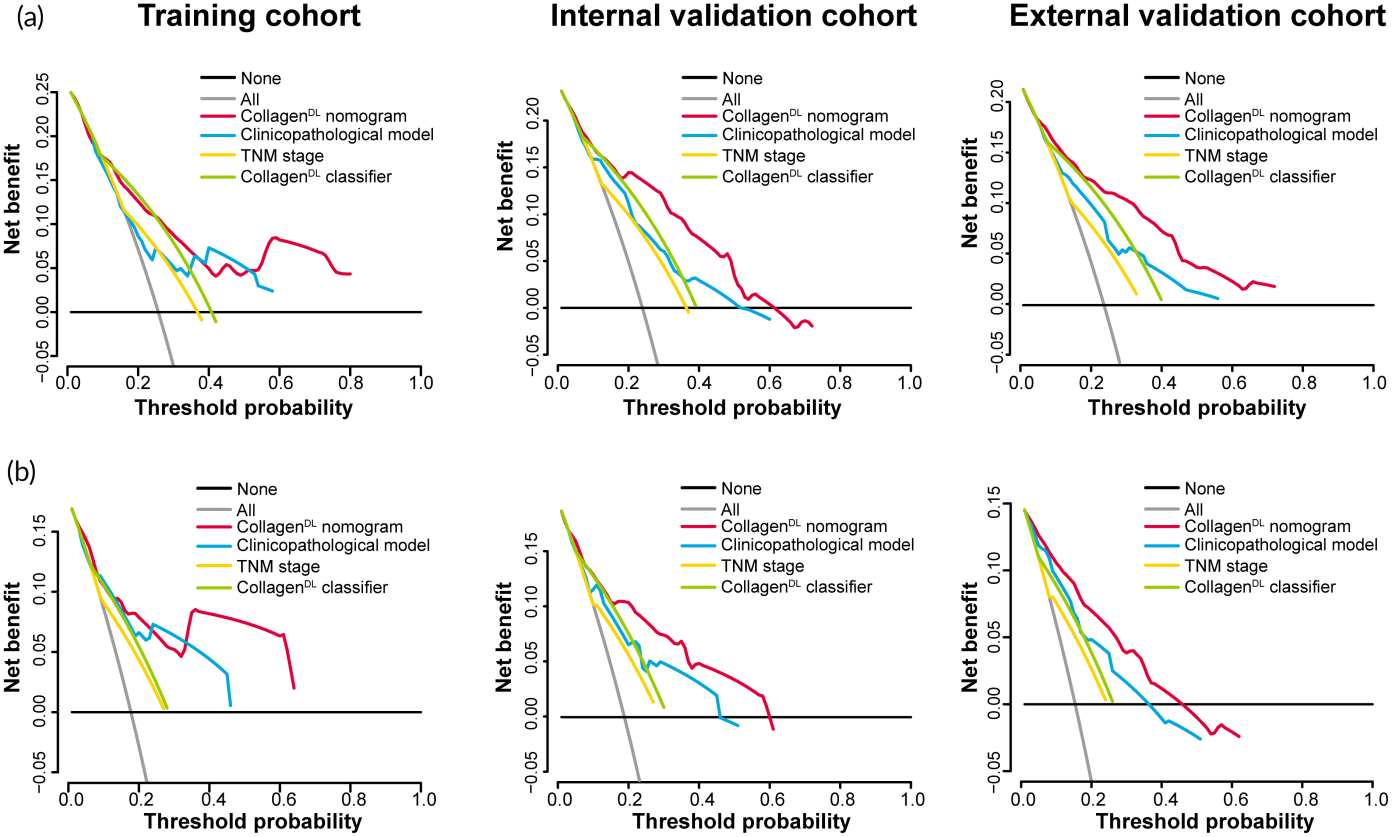
Decision curve analysis for each model in the training, internal validation, and external validation cohorts. Decision curve analysis for DFS (a) and OS (b) in the three cohorts. The y‐axis measures the net benefit, the red line represents the nomogram, the blue line represents the clinicopathological model, the green line represents the collagen^DL^ classifier, the yellow line represents the TNM stage, and the black line and gray line represent the assumptions that none and all of the patients have 5‐year survival. DFS, disease‐free survival; OS, overall survival; TNM, tumor‐node‐metastasis.

Two clinicopathological models based on three clinicopathological predictors in the training cohort were used to predict DFS and OS (Table S3). Compared to either the clinicopathological model, TNM stage, or the collagen^DL^ classifier, the collagen^DL^ nomogram displayed better discrimination with higher C‐indexes in the three cohorts (Table S4). In addition, this result was confirmed by 5‐year time‐dependent ROC curves (Figure S9 and Table S5). The corresponding net reclassification improvement (NRI) and integrated discrimination improvement (IDI) showed that the collagen^DL^ nomogram had a significantly increased classification accuracy for survival outcomes compared with the clinicopathological model (Tables S6 and S7, and Figure 10). Decision curve analysis (DCA) confirmed that the collagen^DL^ nomogram could add more net benefits than the clinicopathological model and TNM stage (Figure 5).

### 2.5 The collagen^DL^ classifier and adjuvant chemotherapy

**FIGURE 6:**
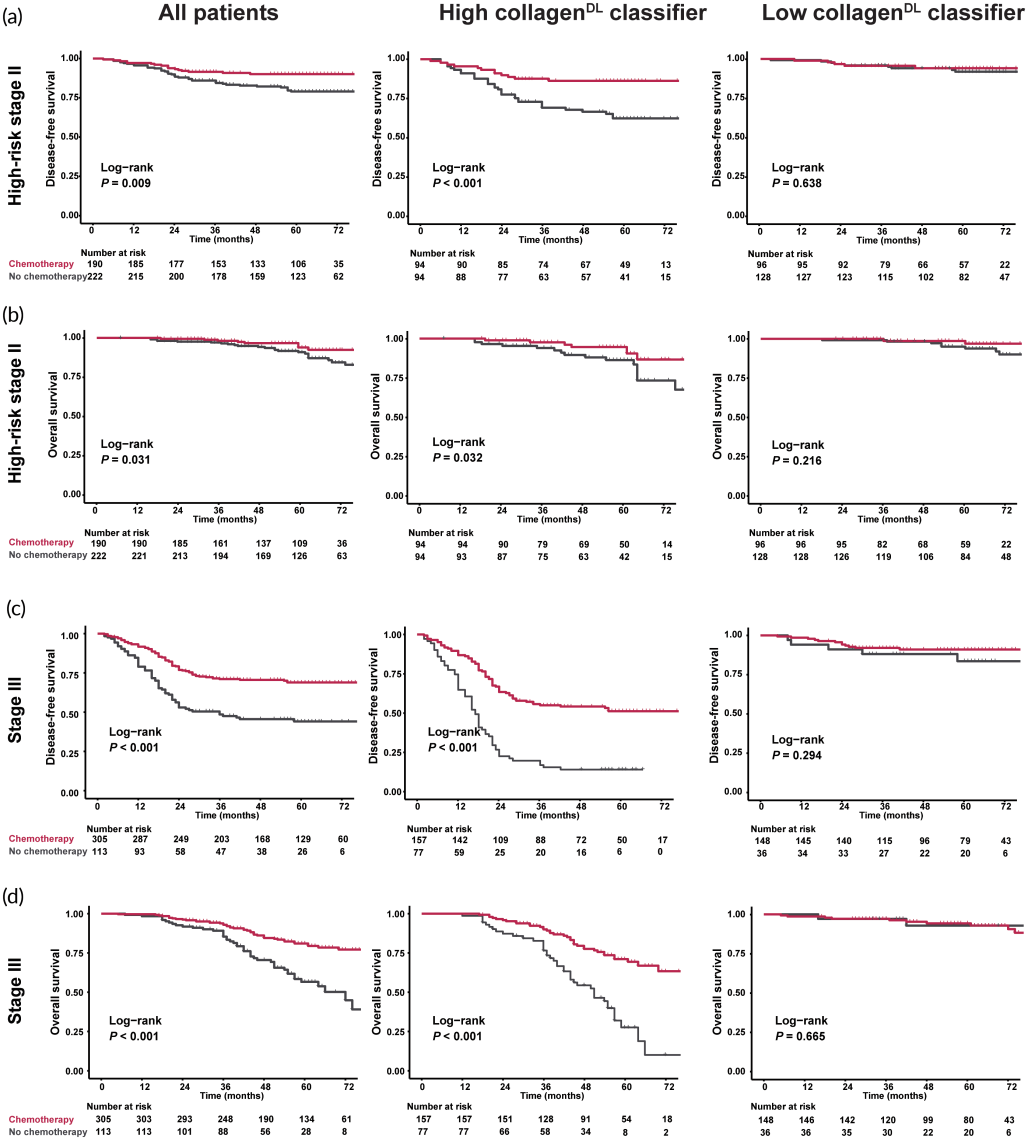
Adjuvant chemotherapy benefits patients with high‐risk stage II and stage III disease in terms of DFS and OS. Adjuvant chemotherapy benefits in patients with high‐risk stage II disease in terms of DFS (a) and OS (b). Adjuvant chemotherapy benefits in patients with stage III disease in terms of DFS (c) and OS (d). On the left is the adjuvant chemotherapy benefits in all patients; in the middle is the adjuvant chemotherapy benefits of the high‐collagen^DL^ classifier group; and on the right is the adjuvant chemotherapy benefits of the low‐collagen^DL^ classifier group. Red represents adjuvant chemotherapy, and black represents no adjuvant chemotherapy after surgery. *P* values were calculated by the log‐rank test. DFS, disease‐free survival; OS, overall survival.

We further analyzed the relationship between the collagen^DL^ classifier and the chemotherapy benefit in high‐risk stage II and III CC patients. The results confirmed that the collagen^DL^ classifier was significantly associated with prognosis regardless of whether patients received chemotherapy (Figure S11). However, the collagen^DL^ classifier might have a more powerful relationship with the prognosis of patients who have not received chemotherapy. Hence, a subset analysis was performed based on the collagen^DL^ classifier. Examination of the interaction between the collagen^DL^ classifier and adjuvant chemotherapy suggested that patients with high collagen^DL^ classifier derived more benefits from adjuvant chemotherapy than patients with low collagen^DL^ classifier in the CC patients with high‐risk II and III stages (Table S8). The corresponding Kaplan–Meier survival curves revealed that adjuvant chemotherapy was significantly associated with improved DFS and OS in the high collagen^DL^ classifier group but had no significant influence in the low collagen^DL^ classifier group (Figure 6).

## 3 DISCUSSION

In the age of precision medicine, individualized prognostic prediction is beneficial for treatment selection and the risk stratification of cancer patients. In this study, we constructed a collagen^DL^ classifier based on multiphoton imaging and a 50‐layer residual network (Res‐net 50) model to improve the prognostic stratification of CC patients with stage II‐III disease after radical resection. Then, two individualized collagen^DL^ nomograms integrating the collagen^DL^ classifier and clinicopathological predictors for DFS and OS were developed, and the collagen^DL^ nomograms achieved satisfactory discrimination and calibration. Moreover, the collagen^DL^ classifier is a potential tool to identify high‐risk stage II and III CC patients who could benefit from adjuvant chemotherapy.

Collagen, as the main component of the ECM, has physiological activity and is responsible for intercellular communication, cell adhesion, and cell proliferation.^6^, ^7^ The structure of collagen can change with the occurrence and development of tumors.^10^, ^22^, ^23^ Collagen is generally curled or smooth in normal epithelial structures. With the development of tumors, the collagen structure gradually thickens, linearizes, hardens, and remolds; hence, collagen structure alterations contain much prognostic information.^24^, ^25^ Zhou and colleagues found that the collagen arrangement was associated with the prognosis of gastric cancer, and collagen width appears to be a powerful collagen feature for estimating 5‐year OS.^10^ Furthermore, the collagen structure is related to chemotherapy resistance. Collagen crosslinking promotes an increase in tissue hardness, which changes the growth and integrity of blood vessels.^26^, ^27^ Excessive deposition and abnormal remodeling of collagen can increase interstitial pressure, which affects the effect of drug delivery.^6^, ^26^, ^27^ Taken together, these pieces of evidence demonstrate that collagen in the TME is a potentially valuable biomarker for estimating prognosis and chemotherapy benefits. The activation map, which can determine the regions that the Res‐net 50 model assigns high values in multiphoton images, suggests that patients with collagen with thicker, linear, and increased crosslinking and larger areas were more likely to have a high collagen^DL^ classifier by the model and have a poor prognosis (Figure 2a).

In addition to its effects on cancer cells, collagen might also impact immune cell infiltration.^28^, ^29^ Of immune cells, T cells are of special interest due to their involvement in tumor progression and serve as prognostic factors in CC.^30^, ^31^ CD4+ and CD8+ T cells are essential components of tumor immunity, with CD4+ T cells enhancing immune responses and promoting CD8+ T‐cell activation, while CD8+ T cells directly kill tumor cells.^32^ Recent studies have demonstrated that collagen density and stiffness in the ECM can impact CD4+ and CD8+ T‐cell migration speed, with higher‐density collagen environments resulting in slower migration than low‐density collagen environments.^28^, ^33^, ^34^ Moreover, the alignment of collagen can augment and accelerate CD8+ T‐cell migration behavior.^35^ These factors influence the path and speed of TILs into or around the tumor bed.^36^ In addition to migration, collagen density also impacts T‐cell activation. T‐cell activation involves the formation of an immunological synapse between a T‐cell and an antigen‐presenting cell; however, this interaction can be significantly reduced in a high‐density collagen environment, impairing T‐cell proliferation, especially that of CD8+ T cells.^37^, ^38^ Furthermore, markers of cytotoxic T‐cell activity were downregulated by a high‐density collagen matrix compared to a low‐density matrix, while markers of tumor‐infiltrating regulatory T cells (Tregs) were upregulated,^36^, ^39^ suggesting that collagen density also impairs cytotoxic activity. Therefore, collagen plays crucial roles in the localization, dynamic behavior, and function of T cells in the TME. Currently, the immunoscore, which quantifies T‐cell infiltration at the tumor center and invasion margin, has important guiding significance for prognosis judgment in patients with CC.^40^, ^41^ Given the regulatory effect of collagen on T cells in the TME, there is a close relationship between collagen and the immunoscore. Overall, the impact of collagen on immune infiltrating cells, especially T cells, such as CD4+, CD8+, cytotoxic T cells, and Tregs, could be another potential reason for the significant association between the collagen^DL^ classifier and survival outcomes. Further research into the role of collagen in T‐cell function and its potential as a therapeutic target for cancer immunotherapy has a promising future.

Multiphoton imaging has been used for real‐time in vivo imaging and optical biopsy due to its label‐free advantages and stability. Multiphoton imaging can visualize the morphology of cells and the structure of tissues at the subcellular level, which is comparable to traditional hematoxylin and eosin (HE) staining.^13^, ^42^ Importantly, due to the endogenous physical properties of collagen, multiphoton imaging can specifically image the collagen structure.^43^, ^44^ The structural information of collagen analyzed from multiphoton images can be used for the diagnosis and prognosis assessment of several diseases.^9^, ^11^, ^45^, ^46^, ^47^ Therefore, multiphoton imaging is a strong tool to assess the association between the collagen structure in the TME and the prognosis of patients with stage II‐III CC.

After obtaining multiphoton images that present the collagen structure, DL is an effective classification method for building a prediction model. Currently, DL has received more attention as a technology of artificial intelligence. Complex structures associated with specific outcomes are automatically extracted directly from medical images by DL, which can eliminate the need for manual feature engineering in traditional methods. Previous studies proved that DL has acquired the performance of human experts in lesion classification and detection.^15^, ^17^, ^19^ Among the deep‐stacked artificial neural networks that have been proposed, Res‐net, which can reduce gradient disappearance and gradient explosion, can perform deeper analyses and has been widely used in the clinic.^48^, ^49^, ^50^ The lack of image data was also solved by transfer learning.^50^, ^51^, ^52^ Therefore, we established a collagen^DL^ classifier based on Res‐net 50 and multiphoton imaging in our work. The patients in the low‐collagen^DL^ classifier and high‐collagen^DL^ classifier groups showed significant differences in 5‐year DFS and OS, and patients with a low collagen^DL^ classifier had a better prognosis than patients with a high collagen^DL^ classifier. Cox regression analysis showed that the collagen^DL^ classifier is an independent predictor of OS and DFS. Then, two individualized collagen^DL^ nomograms were constructed for DFS and OS by incorporating the collagen^DL^ classifier and clinicopathological predictors. Comparing the collagen^DL^ nomograms with the clinicopathological model and TNM stage, we found that the collagen^DL^ nomograms had better prognostic performance (higher C‐indexes and area under the ROC curves and positive NRI and IDI, *P* < 0.05). DCA also showed that the collagen^DL^ nomograms have better clinical application value. These results indicated the prognostic value of the collagen^DL^ nomograms, which could potentially help clinicians determine individualized systemic treatment schemes to improve prognosis.

Adjuvant chemotherapy is a critical component of the standard treatment scheme for CC patients with high‐risk stage II and III disease. However, some patients do not benefit from chemotherapy and suffer from side effects. Therefore, accurately distinguishing patients with different therapeutic responses is necessary to individualize treatment and improve the overall prognosis of patients. We found that high‐risk stage II and III CC patients with a high collagen^DL^ classifier could significantly benefit from chemotherapy, while patients with a low collagen^DL^ classifier were unlikely to benefit from chemotherapy. Hence, the collagen^DL^ classifier could help to better select and manage high‐risk stage II and III CC patients who should receive adjuvant chemotherapy.

There are still some limitations in our work. First, this study was a retrospective multicenter study. Second, the choice of adjuvant chemotherapy was not random but was determined by the patient and the clinician. Therefore, a prospective, large‐sample, multicenter study is needed to verify the robustness of the collagen^DL^ nomograms. Third, the NCCN Colon/Rectal Cancer Panel endorsed universal mismatch repair (MMR) or microsatellite instability (MSI) testing of all patients with a personal history of colon or rectal cancer to identify individuals with Lynch syndrome in 2017. However, this retrospective study was conducted from January 2009 to December 2014, and many patients did not routinely undergo MMR/MSI testing. MSI is very important for the chemotherapy response; however, this retrospective study did not test MSI for all patients between January 2009 and December 2014, which is a limitation of this study.

## 4 CONCLUSIONS

The collagen^DL^ classifier can effectively classify CC patients with stage II‐III disease and increase the predictive value of the TNM staging system. Furthermore, the collagen^DL^ classifier could be a helpful predictive tool to identify patients who are more likely to benefit from adjuvant chemotherapy. The collagen^DL^ nomograms might facilitate the personalized postoperative surveillance and management of stage II‐III CC patients.

## 5 MATERIALS AND METHODS

### 5.1 Study design and patients

Ethics approval was obtained from the institutional review boards of the two academic medical centers: Nanfang Hospital and the Sixth Affiliated Hospital, Sun Yat‐sen University. The requirement for informed consent was waived for this study. The study was conducted following the guidelines of the Declaration of Helsinki.

The data of patients with stage II‐III CC who underwent radical surgery at either of the two participating centers were reviewed in this retrospective study. The inclusion criteria were as follows: (1) patients ≥18 years; (2) American society of Aneshesiologists (ASA) score of 1–3; (3) histologically confirmed as stage II‐III CC according to the 8th edition American Joint Committee on Cancer (AJCC) staging scheme; (4) underwent histologically confirmed R0 resection; (5) availability of complete clinicopathological and follow‐up data; and (6) availability of specimen sections. The exclusion criteria were as follows: (1) patients with stage I or IV disease; (2) ASA score of 4–5; (3) synchronous malignant neoplasms; and (4) patients with preoperative radiotherapy or chemotherapy. Two centers included patients who met the same inclusion and exclusion criteria. A total of 590 patients were recruited from Nanfang Hospital and were divided into a training cohort (303 patients from January 2009 to December 2011) and an internal validation cohort (287 patients from January 2012 to December 2013). Moreover, an independent external validation cohort included 292 patients recruited between January 2013 and December 2013 from the Sixth Affiliated Hospital, Sun Yat‐sen University (Figure S12).

Clinicopathological characteristics included age, sex, bowel obstruction, primary tumor location, preoperative carcinoembryonic antigen (CEA) level, tumor budding, tumor size, tumor differentiation, VELIPI, T stage, N stage, TNM stage, and adjuvant chemotherapy. All patients were restaged based on the 8th edition of the AJCC staging criteria. The primary objective of the study was to construct an effective prediction model to estimate the DFS and OS of stage II‐III CC patients.

### 5.2 Region of interest selection and multiphoton imaging

Formalin‐fixed, paraffin‐embedded samples were sliced into 5‐μm‐thick serial sections for HE staining. Two gastrointestinal pathologists with more than 10 years of experience who were unaware of the prognostic information used a microscope to reassess the invasive area of the tumor on the HE image. When the two pathologists had different opinions, the final decision was made by the director of the Pathology Department. Finally, three regions of interest (ROIs) of 512 μm × 512 μm per section in the invasive region were randomly chosen, and the corresponding regions on the other serial section were used for multiphoton imaging.

Image acquisition for multiphoton imaging was performed with a 100× original magnification objective on another unstained serial section and then compared with HE staining for histologic assessment.^13^, ^53^ More information about the multiphoton imaging system is shown in the Supplementary Methods.

### 5.3 Collagen deep learning classifier construction

Res‐net is a representative deep convolutional neural network (CNN) that is widely applied in the field of target classification.^50^, ^54^ It can effectively avoid degradation problems to train deeper and more powerful networks than previously used networks. In this research, we established the collagen^DL^ classifier based on the Res‐net 50 model with multiphoton imaging in the training cohort. First, multiphoton images were annotated with labels (recurrence or nonrecurrence), and all the multiphoton images were resized to 224 × 224 pixels for input into the model. Data augmentation was utilized to expand the number of images, which included horizontal and vertical flipping, cropping, and scaling of the image. Then, we built a deep CNN prediction model as a feature extractor using the pretrained Res‐net 50 on the ImageNet dataset, which was pretrained on 14 million labeled images from the ImageNet database. The structure of the Res‐net 50 model was kept, in addition to the last fully connected layer, in which the activation function was changed from “ReLU” to “sigmoid” in the output layer. After fine‐tuning the parameters of the Res‐net 50 model, the learning rate, number of epochs, and batch size were set to 0.0001, 100, and 28, respectively. The network training was optimized with the Adam optimizer. The loss function was determined as binary cross‐entropy. Each multiphoton image yielded a probability value of recurrence according to the model, and the average value of three multiphoton images from the section was computed as the final probability value of recurrence. Finally, the patients were classified into low and high collagen^DL^ classifier subgroups based on the optimal cut‐off value, which was determined by the “survminer” R package in the training cohort. The prediction model with the same cut‐off value was used on the internal and external validation cohorts.

The prognostic value of the collagen^DL^ classifier was evaluated through 5‐year time‐dependent ROC curve, Kaplan–Meier survival analysis, and C‐index.^55^

### 5.4 Individualized collagen^DL^ nomogram construction and performance assessment

In the training cohort, univariate Cox regression analysis was used to select candidate predictors for DFS and OS from the collagen^DL^ classifier and clinicopathological characteristics. Variables with *P* < 0.05 were included as candidate predictors in the multivariable Cox regression analysis. Independent prognostic predictors for DFS and OS were identified by multivariable Cox regression analysis with backward stepwise elimination using the Akaike information criterion. Then, two individualized prediction models for predicting DFS and OS were built based on multivariable Cox regression and are presented as nomograms.^56^, ^57^

The discriminative ability and calibration of the collagen^DL^ nomograms were assessed via the C‐index, 5‐year time‐dependent ROC curve, and calibration curve. DCA was applied to determine the clinical application value of the collagen^DL^ nomograms.^58^

### 5.5 Assessment of the incremental value of the collagen^DL^ classifier in individualized DFS and OS estimations

The incremental value of the collagen^DL^ classifier for the clinicopathological model, which was based on clinicopathological predictors, was evaluated with respect to discrimination, calibration, and clinical application value. In addition, the performance of the collagen^DL^ nomogram and the clinicopathological model were compared by the NRI and IDI.^59^, ^60^

### 5.6 Statistical analysis

The Res‐net 50 model was implemented with the open‐source software Python (version 3.9.0) and TensorFlow (version 2.6.0‐GPU), and statistical analysis was conducted in R software (version 3.6.0) and SPSS software (version 22.0). The chi‐square test or Fisher's exact test was used to assess differences between two groups of categorical variables. Univariate and multivariable Cox analyses were used to select the predictors and calculate the HR with 95% CI. The difference between Kaplan–Meier curves was assessed using the log‐rank test. All tests were two‐tailed, and a *P* value <0.050 was determined to be statistically significant.

## AUTHOR CONTRIBUTIONS

**Wei Jiang:** Conceptualization (equal); data curation (equal); formal analysis (equal); investigation (equal); methodology (equal); software (equal); visualization (equal); writing – original draft (equal). **Huaiming Wang:** Conceptualization (equal); data curation (equal); methodology (equal); software (equal); validation (equal); visualization (equal); writing – original draft (equal). **Weisheng Chen:** Conceptualization (equal); data curation (equal); methodology (equal); software (equal); visualization (equal); writing – original draft (equal). **Yandong Zhao:** Conceptualization (equal); data curation (equal); investigation (equal); methodology (equal); visualization (equal); writing – original draft (equal). **Botao Yan:** Data curation (equal); methodology (equal); visualization (equal). **Dexin Chen:** Conceptualization (equal); data curation (equal); software (equal). **Xiaoyu Dong:** Data curation (supporting); formal analysis (supporting); investigation (equal). **Jiaxin Cheng:** Data curation (supporting); formal analysis (supporting); methodology (supporting). **Zexi Lin:** Data curation (supporting); methodology (supporting); software (supporting). **Shuangmu Zhuo:** Conceptualization (lead); funding acquisition (equal); methodology (equal); project administration (lead); software (lead); supervision (lead); visualization (lead); writing – review and editing (lead). **Hui Wang:** Conceptualization (lead); data curation (lead); formal analysis (lead); investigation (lead); methodology (lead); project administration (lead); supervision (lead); writing – review and editing (lead). **Jun Yan:** Conceptualization (lead); data curation (lead); funding acquisition (lead); investigation (lead); methodology (lead); project administration (lead); software (lead); supervision (lead); writing – review and editing (lead).

## CONFLICT OF INTEREST STATEMENT

The authors declare no competing interests.

### PEER REVIEW

The peer review history for this article is available at https://www.webofscience.com/api/gateway/wos/peer-review/10.1002/btm2.10526.

## Supporting information


**Data S1.** Supporting Information.

## Data Availability

The datasets generated and/or analyzed during the current study are available from the corresponding author on reasonable request.
