# Supplementary material for: Association of collagen deep learning classifier with prognosis and chemotherapy benefits in stage II‐III colon cancer
Source: Bioeng Transl Med. 2023 Apr 17;8(3):e10526. doi: 10.1002/btm2.10526 (PMC10189440; doi:10.1002/btm2.10526)
Supplement: Supplementary file 1 — Data S1. Supporting Information. [file BTM2-8-e10526-s001.docx]

Supplementary Materials

[Supplementary Methods 2](#_Toc128771564)

[Multiphoton imaging 2](#_Toc128771565)

[Decision curve analysis. 3](#_Toc128771566)

[Integrated discrimination improvement and net reclassification improvement. 4](#_Toc128771567)

[R software packages used for statistical analysis. 5](#_Toc128771568)

[Supplementary Figures 6](#_Toc128771569)

[Fig. S1. Plots the best cutoff value of the probability value in the training cohort using the K-M method. 6](#_Toc128771570)

[Fig. S2. Time-dependent ROC curves of the collagen^DL^ classifier for DFS and OS. 7](#_Toc128771571)

[Fig. S3. Kaplan–Meier survival analyses of DFS in the training cohort according to the collagen^DL^ classifier stratified by clinicopathological characteristics. 8](#_Toc128771572)

[Fig. S4. Kaplan–Meier survival analysis of OS in the training cohort according to the collagen^DL^ classifier stratified by clinicopathological characteristics. 9](#_Toc128771573)

[Fig. S5. Kaplan–Meier survival analysis of DFS in the internal validation cohort according to the collagen^DL^ classifier stratified by clinicopathological characteristics. 10](#_Toc128771574)

[Fig. S6. Kaplan–Meier survival analysis of OS in the internal validation cohort according to the collagen^DL^ classifier stratified by clinicopathological characteristics. 11](#_Toc128771575)

[Fig. S7. Kaplan–Meier survival analysis of DFS in the external validation cohort according to the collagen^DL^ classifier stratified by clinicopathological characteristics. 12](#_Toc128771576)

[Fig. S8. Kaplan–Meier survival analysis of OS in the external validation according to the collagen^DL^ classifier stratified by clinicopathological characteristics. 13](#_Toc128771577)

[Fig. S9. Time-dependent ROC curves of different models for DFS and OS. 14](#_Toc128771578)

[Fig. S10. Plots of NRI in the training, internal and external validation cohorts. 15](#_Toc128771579)

[Fig. S11. Kaplan–Meier analyses of high-risk stage II and stage III patients with or without adjuvant chemotherapy according to the collagen^DL^ classifier. 16](#_Toc128771580)

[Fig. S12. Flow chart of patient inclusion and exclusion. 17](#_Toc128771581)

[Supplementary Tables 18](#_Toc128771582)

[Table S1. Characteristics of the patients according to the collagen^DL^ classifier in the training and internal and external validation cohorts. 18](#_Toc128771583)

[Table S2. Univariate Cox regression analyses for disease-free and overall survival in the training cohort. 19](#_Toc128771584)

[Table S3. Multivariable Cox regression analyses for disease-free and overall survival without the collagen^DL^ classifier in the training cohort. 20](#_Toc128771585)

[Table S4. C-index comparison of the collagen^DL^ nomogram and other prediction models. 21](#_Toc128771586)

[Table S5. ROC comparison of the collagen^DL^ nomogram and other prediction models at 5 years. 22](#_Toc128771587)

[Table S6. Net reclassification improvement by comparing the collagen^DL^ nomogram with the clinicopathological model. 23](#_Toc128771588)

[Table S7. Integrated discrimination improvement by comparing the collagen^DL^ nomogram with the clinicopathological model. 24](#_Toc128771589)

[Table S8. Adjuvant chemotherapy interaction with the collagen^DL^ classifier for DFS and OS in patients with high-risk stage II and stage III disease. 25](#_Toc128771590)

# Supplementary Methods

**Multiphoton imaging**

Multiphoton imaging was completed by an optical researcher. The nonlinear optical microscope was constructed using a commercial laser scanning microscope (LSM 880 Zeiss, Germany) and a mode-locked titanium:sapphire femtosecond laser (140 fs, 80 MHz). The microscope can be adjusted in the spectrum from 680 to 1080 nm. The excitation laser with a flat apochromatic objective (10×, numerical aperture, NA = 0.8) was concentrated on the tumor sample. The two-channel mode was performed to acquire two-photon excitation fluorescence (TPEF) and second harmonic generation (SHG) signals. One channel corresponding to the wavelength range between 430 and 708 nm displays the morphologies of tissue components from the TPEF signals (color with green), whereas another channel covering the wavelength range between 387 and 409 nm presents the structure of collagen components from the SHG signals (color with red). Then, the multiphoton image was compared with the HE image for histologic assessment.

## Decision curve analysis.

Decision curve analysis (DCA) was used to quantitatively analyze the net benefit at different threshold probabilities to evaluate the clinical usefulness of the prediction model classifier. DCA is based on the principle that the relative loss value of false positives and false negatives can be expressed by a threshold probability. The value of the threshold probability can determine the clinical outcome of subjects, and the clinical net benefit (NB) function can be used to model the clinical results of true positives and false positives:

$$\text{NB=}\frac{\text{true}\text{ }\text{positive}}{\text{n}}\text{-}\frac{\text{false}\text{ }\text{positive}}{\text{n}}\text{×}\frac{\text{P}_{\text{t}}}{\text{1-}\text{P}_{\text{t}}}$$

where n is the sample size, Pt is the threshold probability, and true positive and false positive are the number of true positive and false positive cases, respectively. NB balances the relationship between the gain value and the loss value based on this theory. If the subject's NB is within the acceptable range, intervention or corresponding treatment measures will be recommended. The generation of the DCA curve changes the size of NB through a series of threshold probability changes.

## Integrated discrimination improvement and net reclassification improvement.

Integrated discrimination improvement (IDI) and net reclassification improvement (NRI) are used to compare the diagnostic capabilities of two prediction models and whether one model improves the diagnostic accuracy of the other. Therefore, NRI and IDI are receiving more attention from experts in research. The calculation formulas of IDI and NRI are as follows:

IDI =（IS_new_ – IS_old_）-（IP_new_ – IP_old_）

NRI =（IS_new_- IS_old_）+（IP_new_ - IP_old_）

IS: Integral sensitivity over all possible cutoff values

IP: 1-specificity under different classification threshold settings

The larger the IDI or NRI is, the better the prediction ability of the new model. If IDI or NRI > 0, it is a positive improvement, indicating that the prediction ability of the new model is improved compared with the old model. If IDI or NRI < 0, it is considered that the prediction ability of the new model is decreased compared with the old model. If IDI or NRI = 0, it is considered that the new model is not improved.

## R software packages used for statistical analysis.

Survival analysis was performed with the “survival” package. The optimal cutoff value of high- and low-collagen^DL^ classifier was defined by the “survminer” R package. according to the Nomograms and calibration plots were generated with the “rms” package. A time-dependent receiver operating characteristic (ROC) curve was generated with the “survivalROC” package. Comparisons between ROC curves were performed with the “timeROC” package. The “survIDINRI” package was used for the calculation of NRI and IDI. DCA was performed with the function of the “dca.R” package. The reported statistical significance levels were all two-sided. The statistical significance level was set at 0.05.

# Supplementary Figures


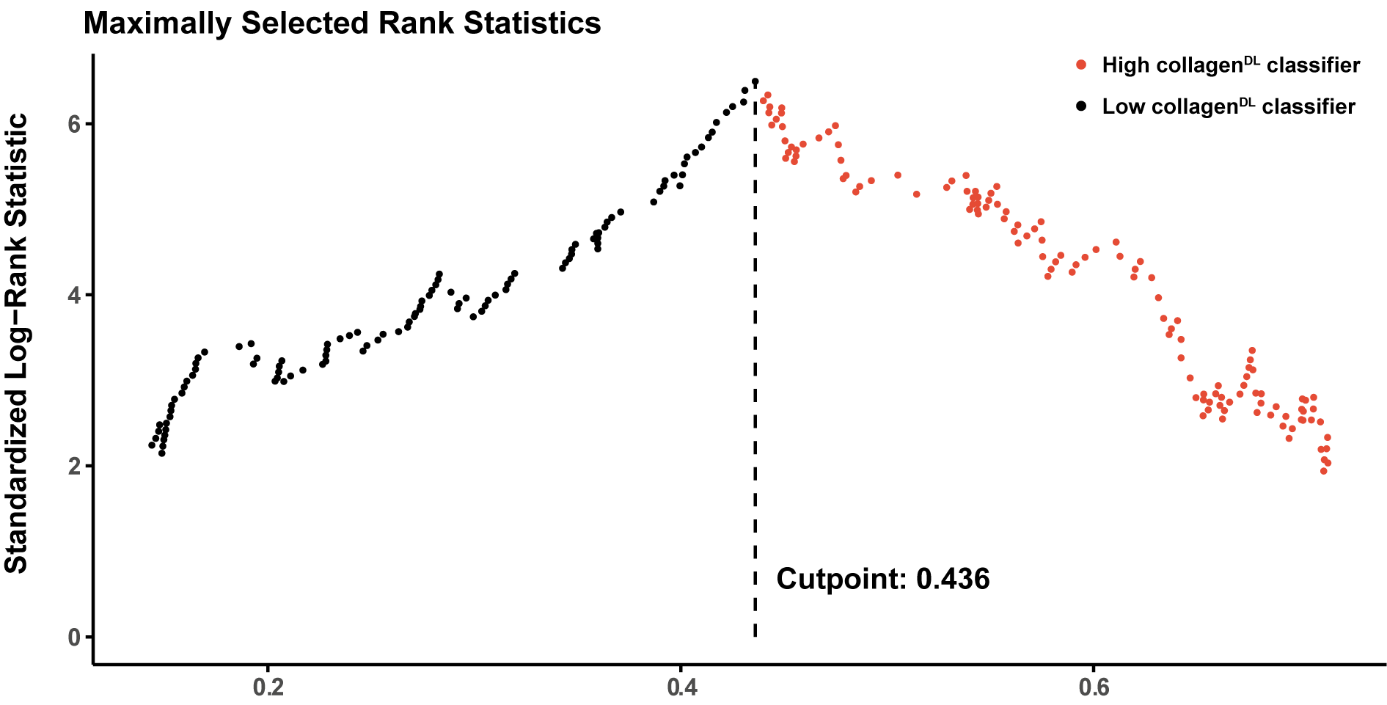


## Fig. S1. Plots the best cutoff value of the probability value in the training cohort using the K-M method.


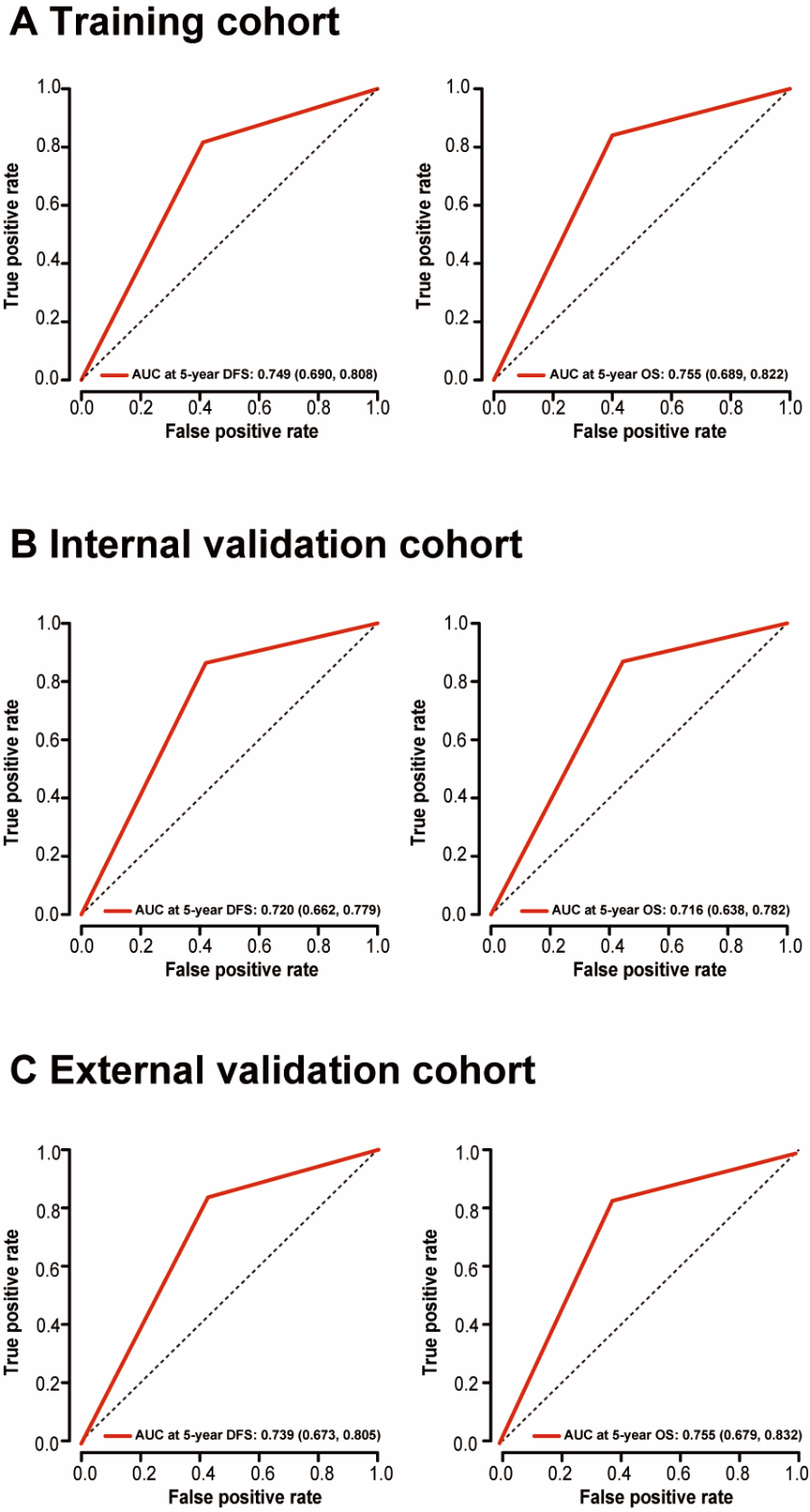


**Fig. S2. Time-dependent ROC curves of the collagen^DL^ classifier for DFS and OS.**

Time-dependent ROC curve for the collagen^DL^ classifier as predictors of 5 years in the training **(A)**, internal validation **(B)**, and external validation cohorts **(C)**. Left: ROC curves for DFS; Right: ROC carvers for OS. *Abbreviations*: ROC, receiver operator characteristic curve; AUC, area under the receiver operator characteristic curve; DFS, disease-free survival; OS, overall survival.


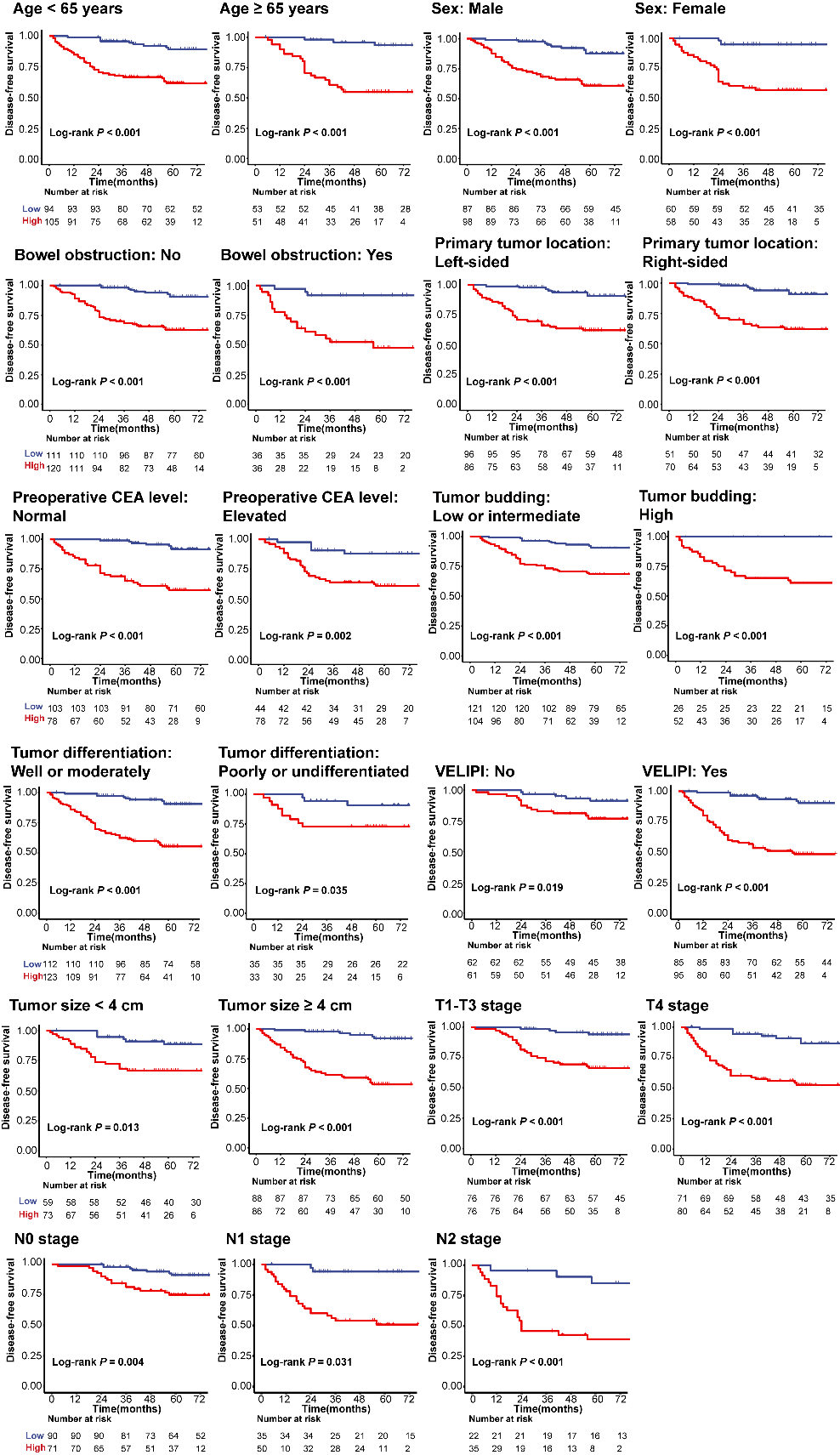


## **Fig. S3. Kaplan–Meier survival analyses of DFS in the training cohort according to the collagen^DL^ classifier stratified by clinicopathological characteristics.**

*P* values were calculated by the log-rank test.


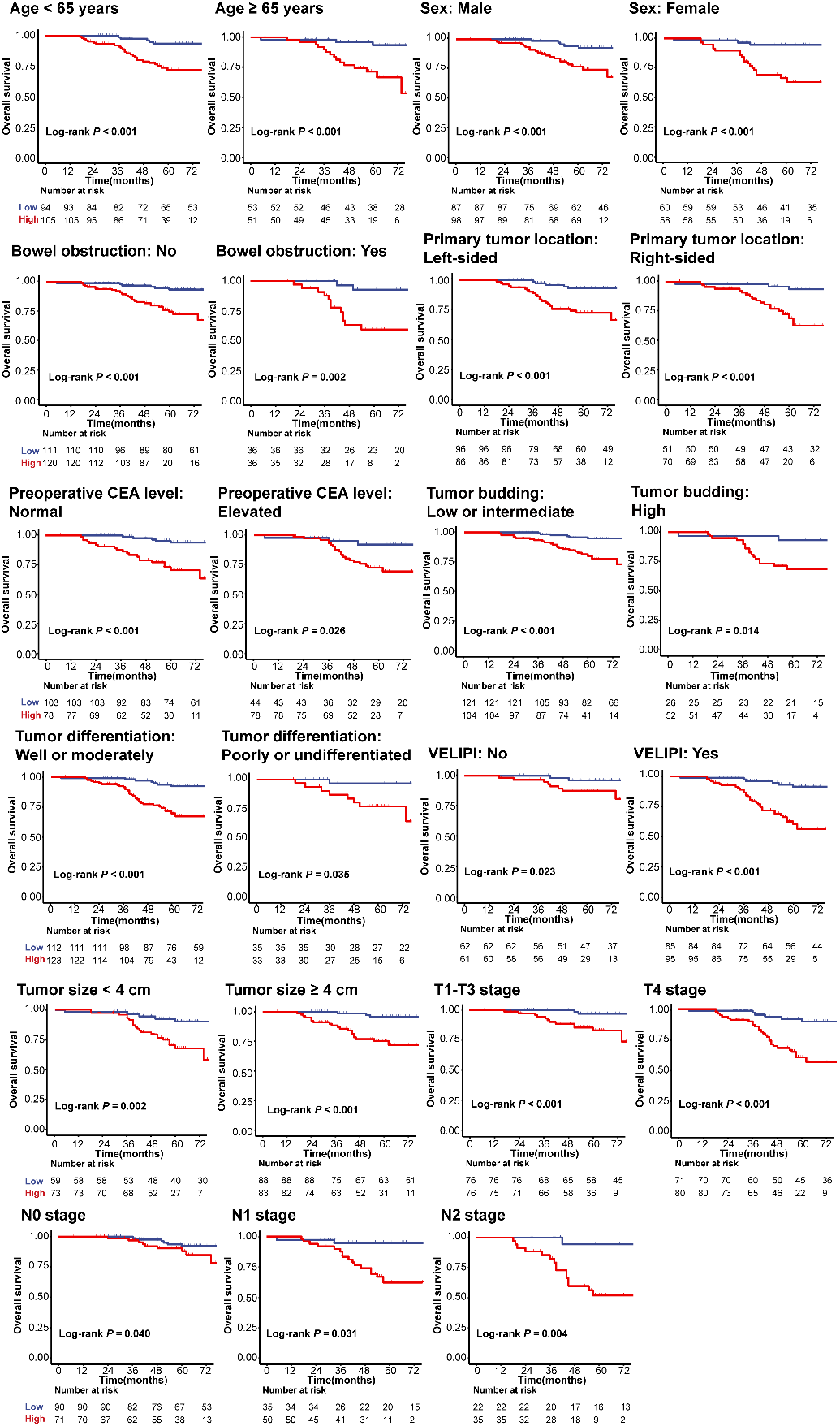


## **Fig. S4. Kaplan–Meier survival analysis of OS in the training cohort according to the collagen^DL^ classifier stratified by clinicopathological characteristics.**

*P* values were calculated by the log-rank test.


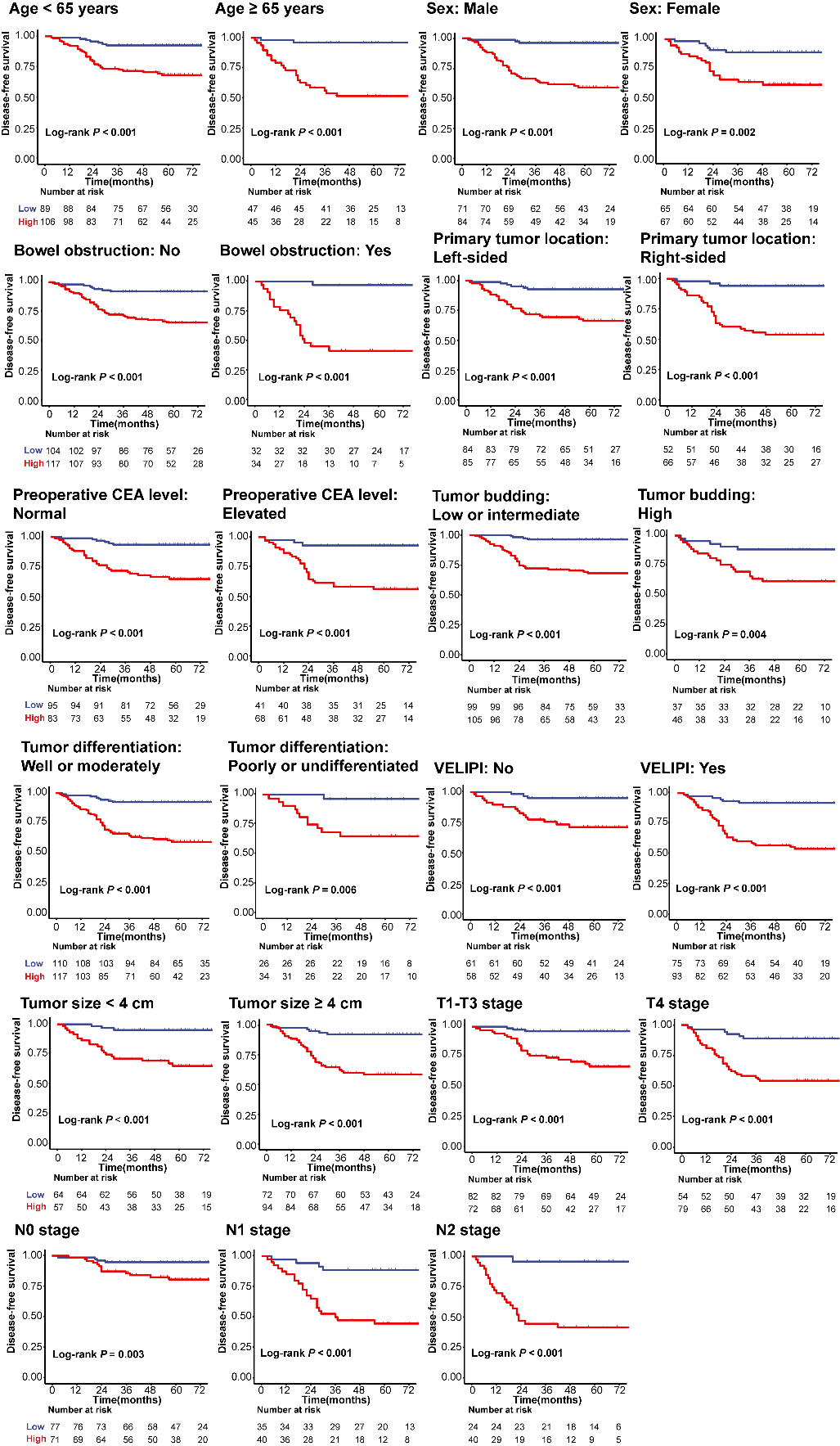


## Fig. S5. Kaplan–Meier survival analysis of DFS in the internal validation cohort according to the collagen^DL^ classifier stratified by clinicopathological characteristics.

*P* values were calculated by the log-rank test.


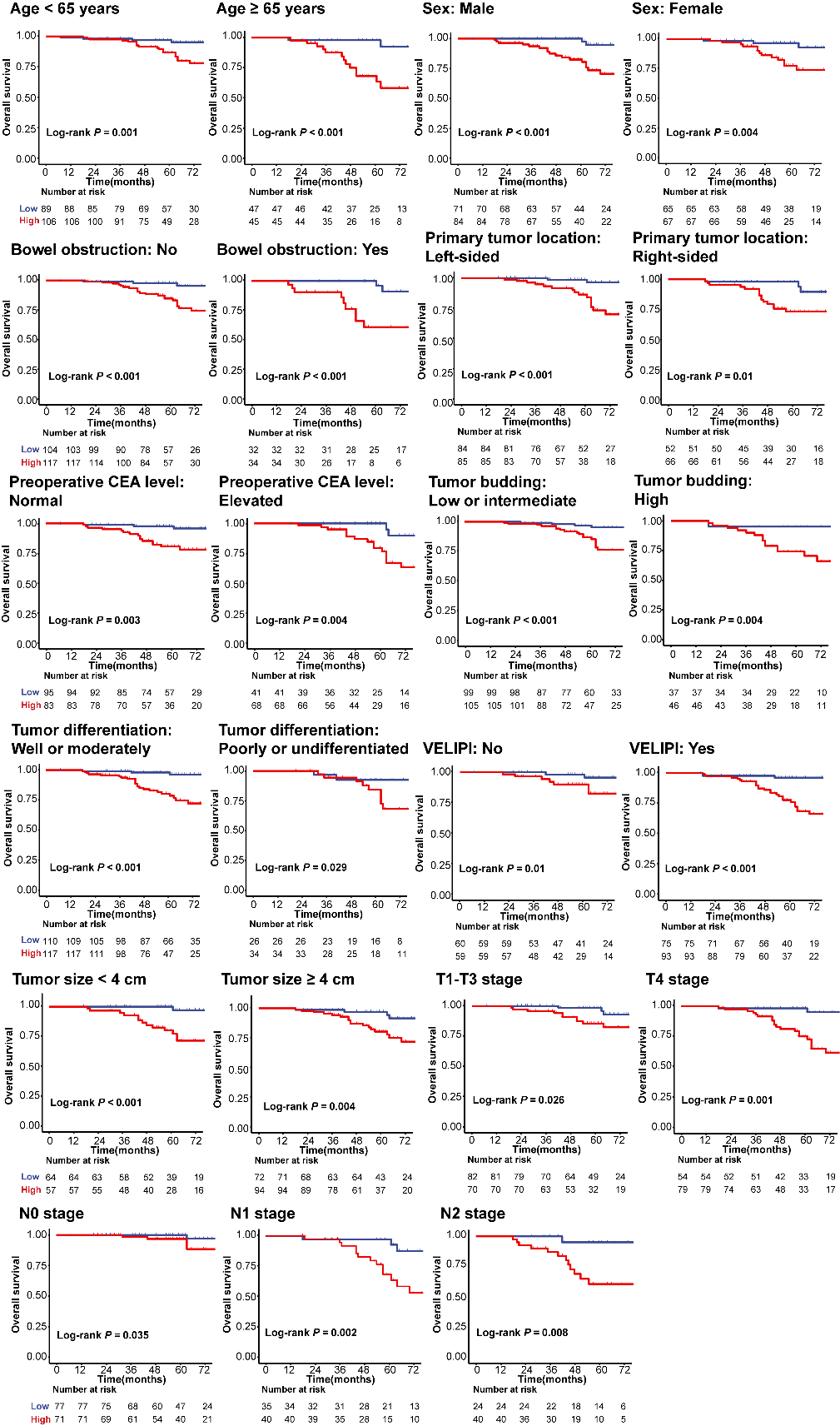


## Fig. S6. Kaplan–Meier survival analysis of OS in the internal validation cohort according to the collagen^DL^ classifier stratified by clinicopathological characteristics.

*P* values were calculated by the log-rank test.


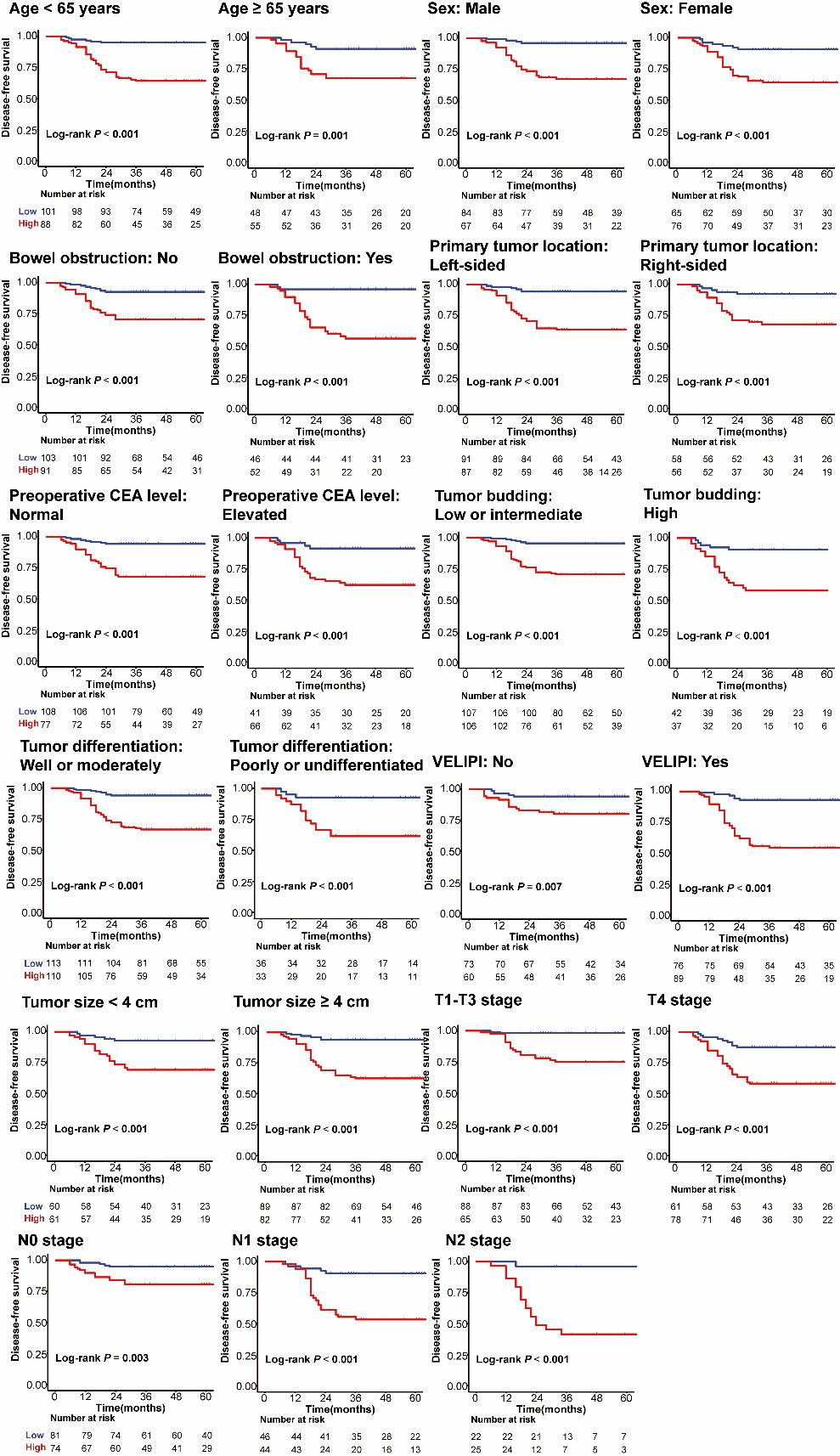


## Fig. S7. Kaplan–Meier survival analysis of DFS in the external validation cohort according to the collagen^DL^ classifier stratified by clinicopathological characteristics.

*P* values were calculated by the log-rank test.

**
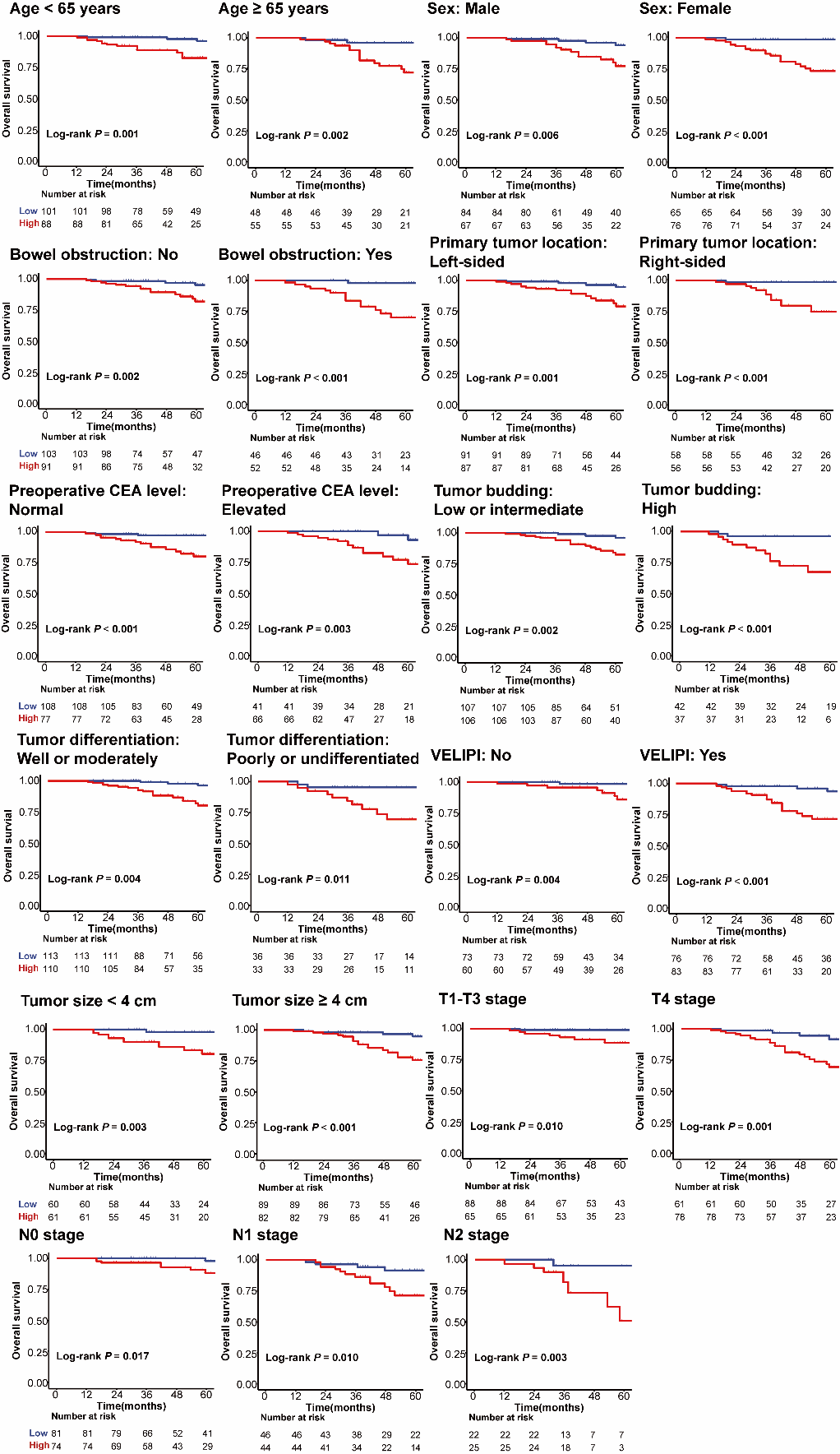
**

## Fig. S8. Kaplan–Meier survival analysis of OS in the external validation according to the collagen^DL^ classifier stratified by clinicopathological characteristics.

*P* values were calculated by the log-rank test.


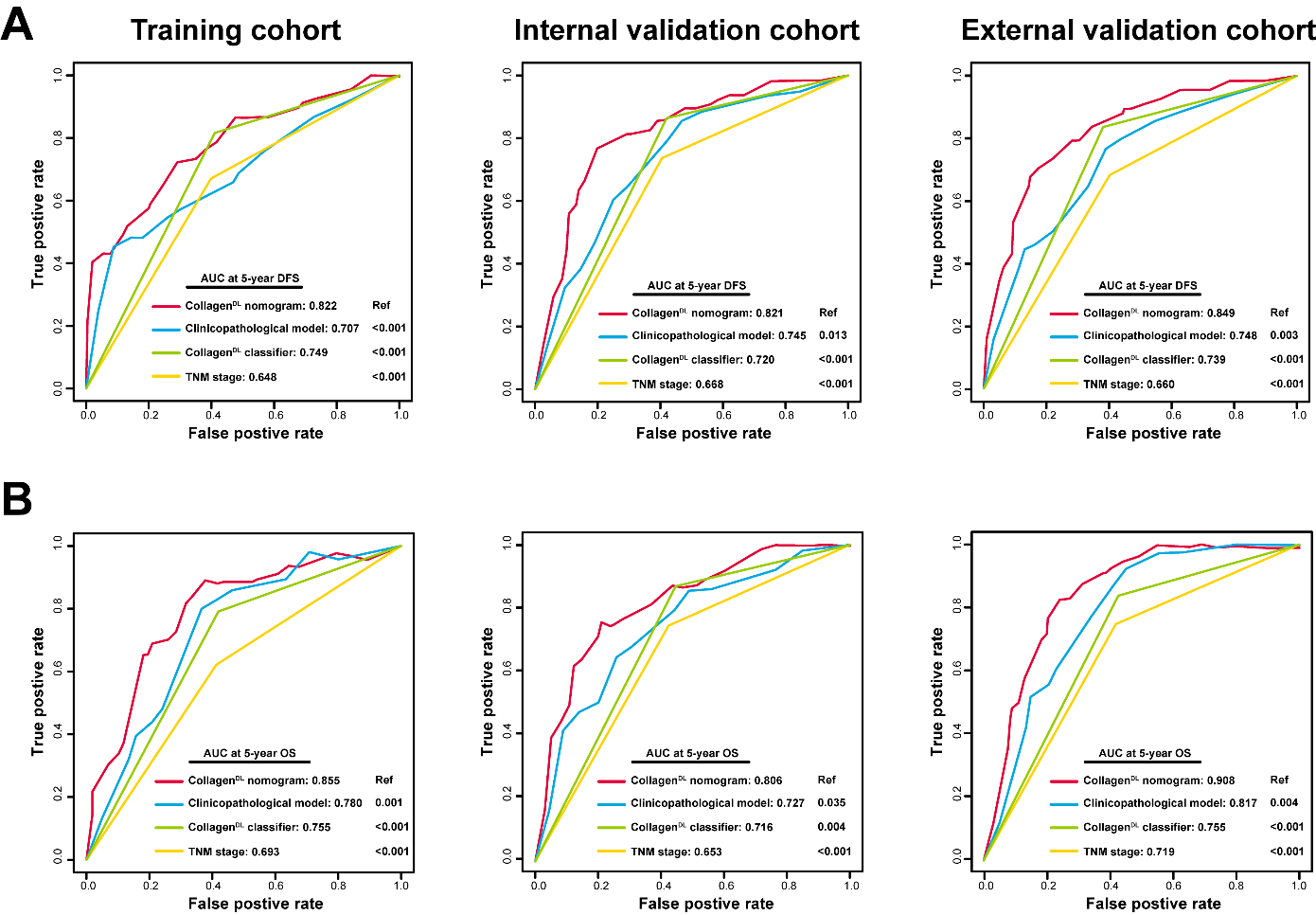


Fig. S9. Time-dependent ROC curves of different models for DFS and OS.

Time-dependent ROC curves of different models for DFS **(A)** and OS **(B)** in the training, internal and external validation cohorts. *Abbreviations:* DFS, disease-free survival; OS, overall survival; TNM, tumor-node-metastasis; ROC, receiver operator characteristic curve; AUC, area under the receiver operator characteristic curve.


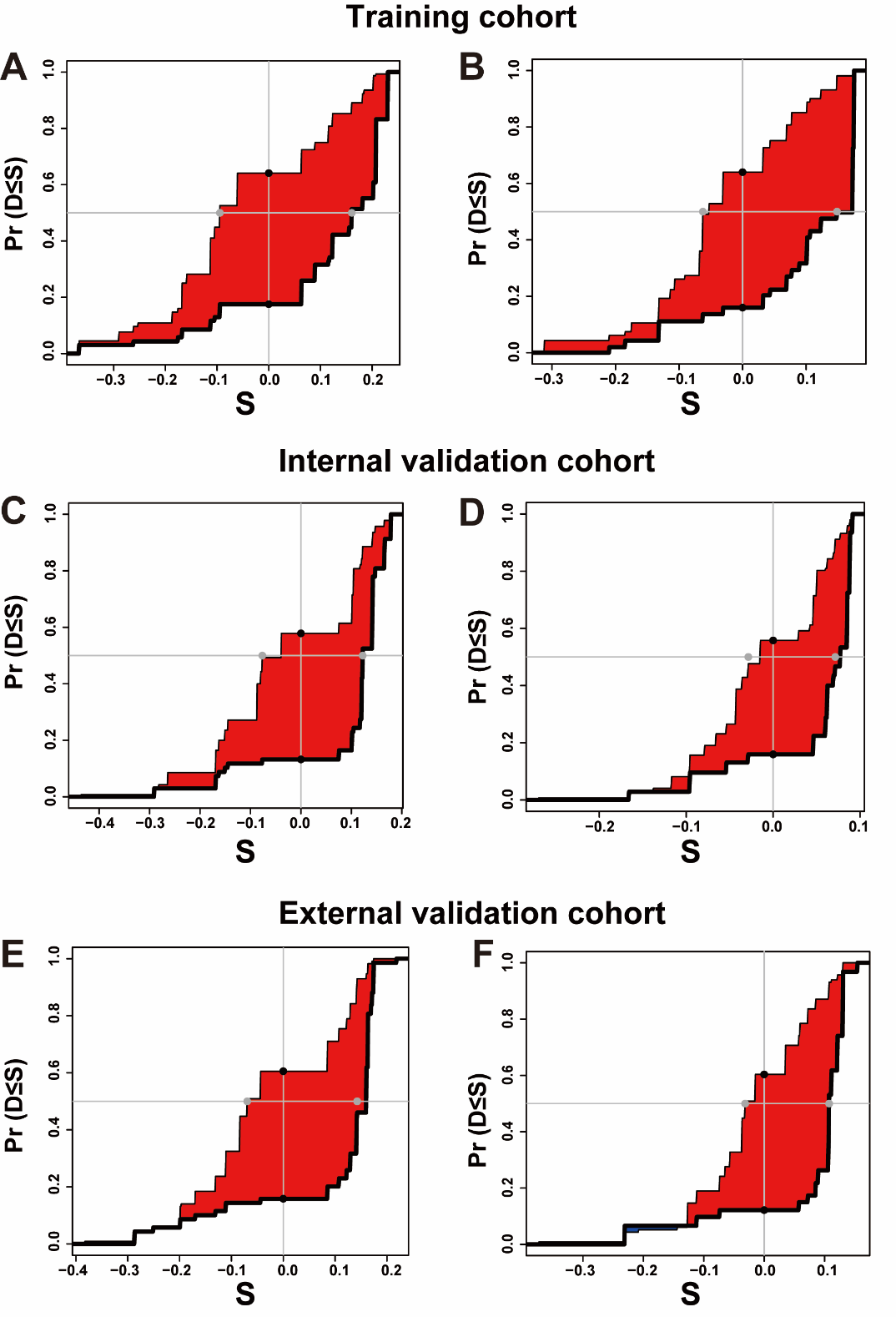


## Fig. S10. Plots of NRI in the training, internal and external validation cohorts.

Net reclassification improvement by comparing the collagen^DL^ nomogram with the clinicopathological model in the training, internal and external validation cohorts. **(A) (C) (E)** for DFS; **(B) (D) (F)** for OS. *Abbreviations:* DFS, disease-free survival; OS, overall survival; NRI, net reclassification improvement.

**
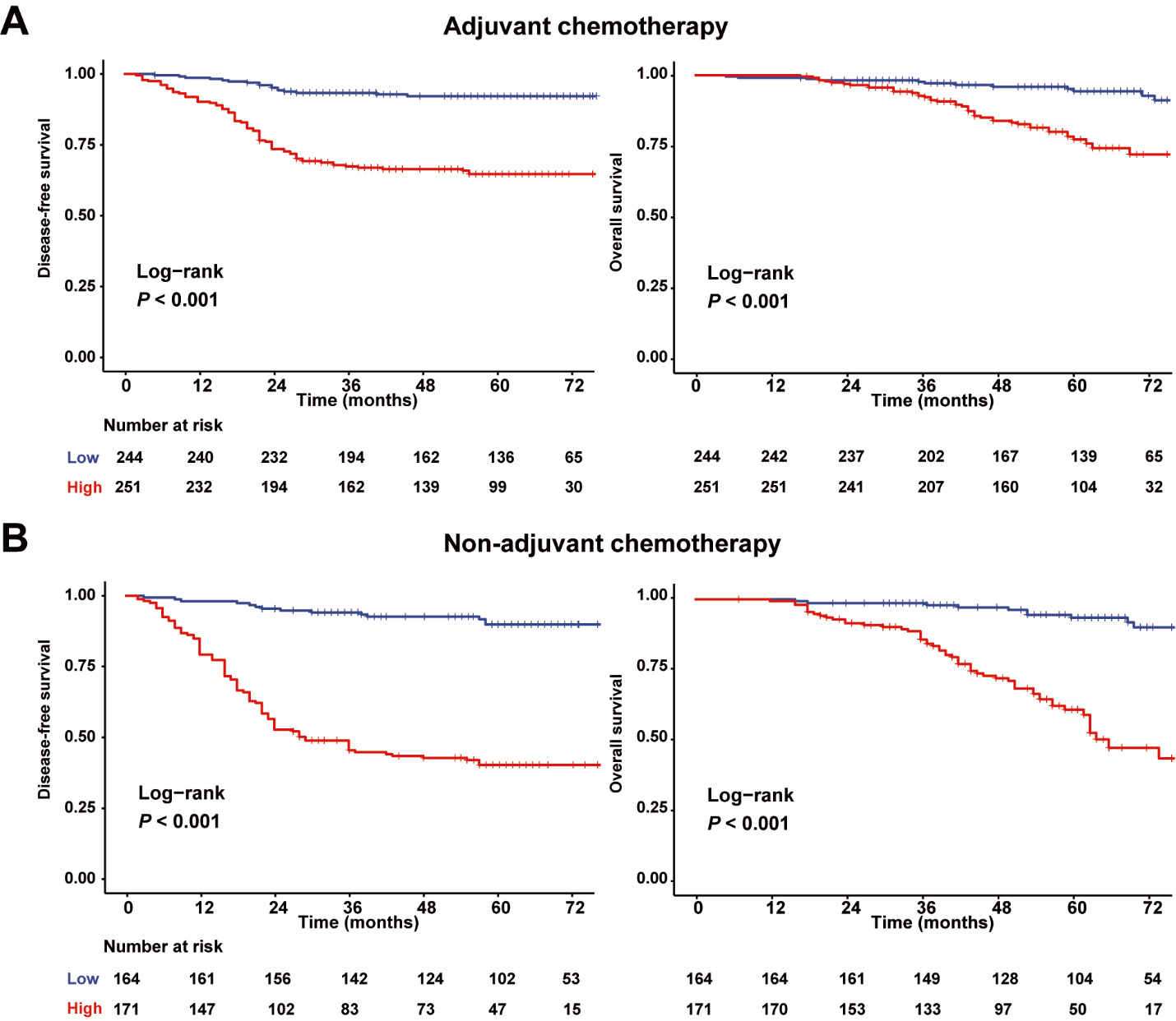
**

## Fig. S11. Kaplan–Meier analyses of high-risk stage II and stage III patients with or without adjuvant chemotherapy according to the collagen^DL^ classifier.

**(A)** Kaplan–Meier survival analyses of high-risk stage II and stage III patients with adjuvant chemotherapy. **(B)** Kaplan–Meier survival analyses of high-risk stage II and stage III patients without adjuvant chemotherapy. *P* values was calculated by the log-rank test.

**
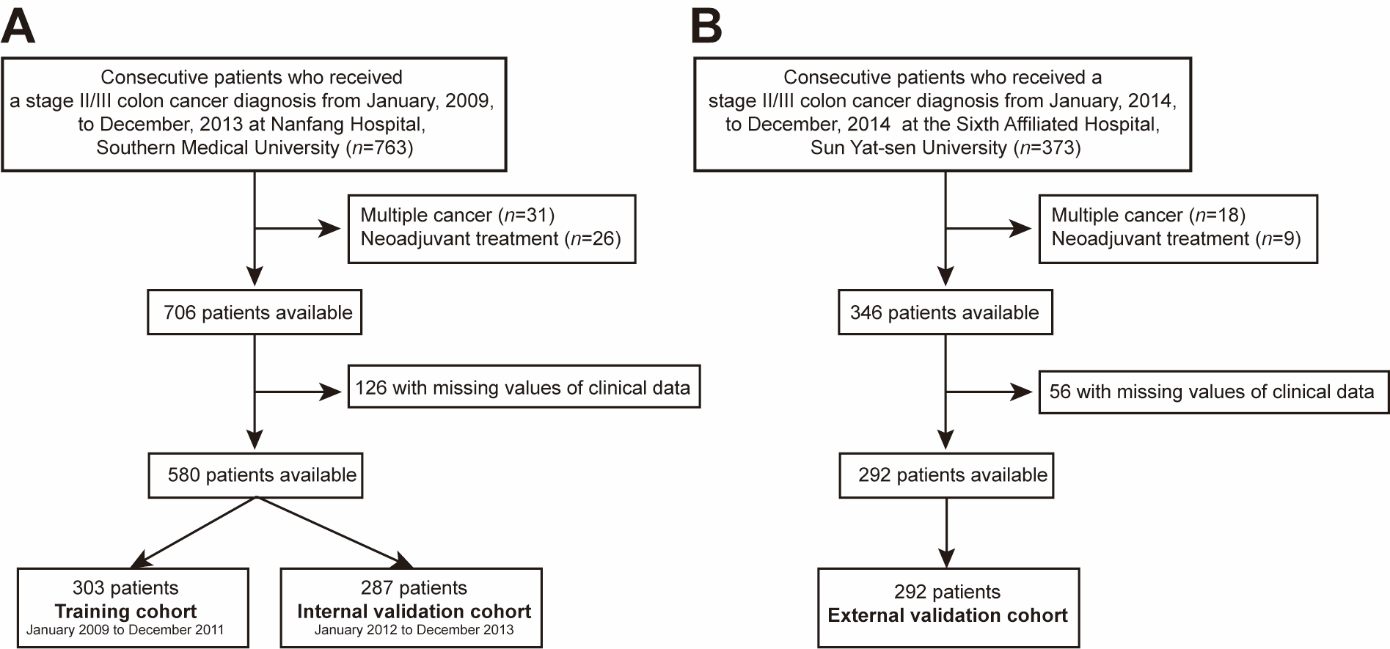
**

## Fig. S12. Flow chart of patient inclusion and exclusion.

# Supplementary Tables

## Table S1. Characteristics of the patients according to the collagen^DL^ classifier in the training and internal and external validation cohorts.

| **Characteristic** | **Training cohort (*n*=303)** | | ***P*** | **Internal validation**  **cohort (*n*=287)** | | ***P*** | **External validation cohort (*n*=292)** | | ***P*** |
| --- | --- | --- | --- | --- | --- | --- | --- | --- | --- |
|  | **Low-collagen^DL^**  ***n*=147** | **High-collagen^DL^**  ***n*=156** |  | **Low-collagen^DL^**  ***n*=136** | **High-collagen^DL^**  ***n*=151** |  | **Low-collagen^DL^**  ***n*=149** | **High-collagen^DL^**  ***n*=143** |  |
| **Age, years old, No. (%)** |  |  | 0.538 |  |  | 0.342 |  |  | 0.264 |
| <65 | 94 (63.9) | 105 (67.3) |  | 89 (65.4) | 106 (70.2) |  | 101 (67.8) | 88 (61.5) |  |
| ≥65 | 53 (36.1) | 51 (32.7) |  | 47 (34.6) | 45 (29.8) |  | 48 (32.2) | 55 (38.5) |  |
| **Sex** |  |  | 0.516 |  |  | 0.561 |  |  | 0.104 |
| Male | 87 (59.2) | 98 (62.8) |  | 71 (52.2) | 84 (55.6) |  | 84 (56.4) | 67 (46.9) |  |
| Female | 60 (40.8) | 58 (37.2) |  | 65 (47.8) | 67 (44.4) |  | 65 (43.6) | 76 (53.1) |  |
| **Bowel obstruction, No. (%)** |  |  | 0.773 |  |  | 0.839 |  |  | 0.321 |
| No | 111 (75.5) | 120 (76.9) |  | 104 (76.5) | 117 (77.5) |  | 103 (69.1) | 91 (63.6) |  |
| Yes | 36 (24.5) | 36 (2301) |  | 32 (23.5) | 34 (22.5) |  | 46 (30.9) | 52 (36.4) |  |
| **Primary tumor location, No. (%)** |  |  | 0.071 |  |  | 0.347 |  |  | 0.967 |
| Left-sided | 96 (65.3) | 86 (55.1) |  | 84 (61.8) | 85 (56.3) |  | 91 (61.1) | 87 (60.8) |  |
| Right-sided | 51 (34.7) | 70 (44.9) |  | 52 (38.2) | 66 (43.7) |  | 58 (38.9) | 56 (39.2) |  |
| **Preoperative CEA level, No. (%)** |  |  | <0.001 |  |  | 0.009 |  |  | 0.001 |
| Normal | 103 (70.1) | 78 (50.0) |  | 95 (69.9) | 83 (55.0) |  | 108 (72.5) | 77 (53.8) |  |
| Elevated | 44 (29.9) | 78 (50.0) |  | 41 (30.1) | 68 (45.0) |  | 41 (27.5) | 66 (46.2) |  |
| **Tumor budding, No. (%)** |  |  | <0.001 |  |  | 0.543 |  |  | 0.656 |
| Low or intermediate | 121 (82.3) | 104 (66.7) |  | 99 (72.8) | 105 (69.5) |  | 107 (71.8) | 106 (74.1) |  |
| High | 26 (17.7) | 52 (33.3) |  | 37 (27.2) | 46 (30.5) |  | 42 (28.2) | 37 (25.9) |  |
| **Tumor differentiation, No. (%)** |  |  | 0.579 |  |  | 0.480 |  |  | 0.827 |
| Well or moderately | 112 (76.2) | 123 (78.8) |  | 110 (80.9) | 117 (77.5) |  | 113 (75.8) | 110 (76.9) |  |
| Poorly or undifferentiated | 35 (26.8) | 33 (21.1) |  | 26 (19.1) | 34 (22.5) |  | 36 (24.2) | 33 (23.1) |  |
| **VELIPI, No. (%)** |  |  | 0.586 |  |  | 0.269 |  |  | 0.228 |
| No | 62 (42.2) | 61 (39.1) |  | 61 (44.9) | 58 (38.4) |  | 73 (49.2) | 60 (42.0) |  |
| Yes | 85 (57.8) | 95 (60.9) |  | 75 (55.1) | 93 (61.6) |  | 76 (51.0) | 83 (58.0) |  |
| **Tumor size, cm, No. (%)** |  |  | 0.243 |  |  | 0.111 |  |  | 0.679 |
| <4 | 59 (40.1) | 73 (46.8) |  | 64 (47.1) | 57 (37.7) |  | 60 (40.3) | 61 (42.7) |  |
| ≥4 | 88 (59.9) | 83 (53.2) |  | 72 (52.9) | 94 (62.3) |  | 89 (59.7) | 82 (57.3) |  |
| **T stage, No. (%)** |  |  | 0.604 |  |  | 0.032 |  |  | 0.020 |
| T1-T3 | 76 (51.7) | 76 (48.7) |  | 82 (60.3) | 72 (47.7) |  | 88 (59.1) | 65 (45.5) |  |
| T4 | 71 (48.3) | 80 (51.3) |  | 54 (39.7) | 79 (52.3) |  | 61 (40.9) | 78 (54.5) |  |
| **N stage, No. (%)** |  |  | 0.022 |  |  | 0.149 |  |  | 0.807 |
| N0 | 90 (61.2) | 71 (45.5) |  | 77 (56.6) | 71 (47.0) |  | 81 (54.4) | 74 (51.7) |  |
| N1 | 35 (23.8) | 50 (32.1) |  | 35 (25.7) | 40 (26.5) |  | 46 (30.9) | 44 (30.8) |  |
| N2 | 22 (15.0) | 35 (22.4) |  | 24 (17.6) | 40 (26.5) |  | 22 (14.8) | 25 (17.5) |  |
| **TNM stage, No. (%)** |  |  | 0.006 |  |  | 0.104 |  |  | 0.655 |
| II | 90 (61.2) | 71 (45.5) |  | 77 (56.6) | 71 (47.0) |  | 81 (54.4) | 74 (51.7) |  |
| III | 57 (38.8) | 85 (54.5) |  | 59 (43.4) | 80 (53.0) |  | 68 (45.6) | 69 (48.3) |  |

Note. Values in parentheses are percentages unless indicated otherwise.

*Abbreviations*: CEA, carcinoembryonic antigen; VELIPI, venous emboli and/or lymphatic invasion and/or perineural invasion; TNM, tumor-node-metastasis.

Table S2. Univariate Cox regression analyses for disease-free and overall survival in the training cohort.

| **Characteristic** | **Disease-free survival** | | **Overall survival** | |
| --- | --- | --- | --- | --- |
|  | **HR (95% CI)** | ***P*** | **HR (95% CI)** | ***P*** |
| **Age, years old** |  |  |  |  |
| <65 | 1 [Reference] |  | 1 [Reference] |  |
| ≥65 | 1.007 (0.625, 1.624) | 0.978 | 1.219 (0.695, 2.139) | 0.490 |
| **Sex** |  |  |  |  |
| Male | 1 [Reference] |  | 1 [Reference] |  |
| Female | 0.977 (0.611, 1.563) | 0.922 | 1.382 (0.797, 2.396) | 0.249 |
| **Bowel obstruction** |  |  |  |  |
| No | 1 [Reference] |  | 1 [Reference] |  |
| Yes | 1.415 (0.854, 2.347) | 0.178 | 1.344 (0.726, 2.488) | 0.347 |
| **Primary tumor location** |  |  |  |  |
| Left-sided | 1 [Reference] |  | 1 [Reference] |  |
| Right-sided | 1.359 (0.861, 2.145) | 0.188 | 1.175 (0.676. 2.040) | 0.567 |
| **Preoperative CEA level** |  |  |  |  |
| Normal | 1 [Reference] |  | 1 [Reference] |  |
| Elevated | 1.420 (0.900, 2.242) | 0.132 | 1.574 (0.907, 2.732) | 0.107 |
| **Tumor budding** |  |  |  |  |
| Low or intermediate | 1 [Reference] |  | 1 [Reference] |  |
| High | 1.538 (0.945, 2.502) | 0.083 | 1.844 (1.045, 3.255) | 0.035 |
| **Tumor differentiation** |  |  |  |  |
| Well or moderately | 1 [Reference] |  | 1 [Reference] |  |
| Poorly or undifferentiated | 1.571 (0.847, 2.916) | 0.152 | 1.119 (0.574, 2.182) | 0.742 |
| **VELIPI** |  |  |  |  |
| No | 1 [Reference] |  | 1 [Reference] |  |
| Yes | 2.315 (1.373, 3.901) | 0.002 | 2.637 (1.380, 5.040) | 0.003 |
| **Tumor size, cm** |  |  |  |  |
| <4 | 1 [Reference] |  | 1 [Reference] |  |
| ≥4 | 1.147 (0.721, 1.825) | 0.562 | 1.318 (0.761, 2.283) | 0.325 |
| **T stage** |  |  |  |  |
| T1-T3 | 1 [Reference] |  | 1 [Reference] |  |
| T4 | 1.811 (1.135, 2.889) | 0.013 | 2.548 (1.409, 4.607) | 0.002 |
| **N stage** |  |  |  |  |
| N0 | 1 [Reference] |  | 1 [Reference] |  |
| N1 | 2.543 (1.459, 4.432) | 0.001 | 2.581 (1.300, 5.124) | 0.007 |
| N2 | 3.567 (2.025, 6.286) | <0.001 | 3.758 (1.915, 7.376) | <0.001 |
| **Collagen^DL^ classifier** |  |  |  |  |
| Low | 1 [Reference] |  | 1 [Reference] |  |
| High | 6.051 (3.259, 11.236) | <0.001 | 5.497 (2.684, 11.258) | <0.001 |

*Abbreviations*: HR, hazard ratio; CI, confidence interval; CEA, carcinoembryonic antigen; VELIPI, venous emboli and/or lymphatic invasion and/or perineural invasion; NA, not available.

## Table S3. Multivariable Cox regression analyses for disease-free and overall survival without the collagen^DL^ classifier in the training cohort.

| **Characteristic** | **Disease-free survival** | | **Overall survival** | |
| --- | --- | --- | --- | --- |
|  | **HR (95% CI)** | ***P*** | **HR (95% CI)** | ***P*** |
| **VELIPI** |  |  |  |  |
| No | 1 [Reference] |  | 1 [Reference] |  |
| Yes | 2.888 (1.183, 4.394) | 0.002 | 2.059 (1.216, 3.487) | 0.009 |
| **T stage** |  |  |  |  |
| T1-T3 | 1 [Reference] |  | 1 [Reference] |  |
| T4 | 1.762 (1.099, 2.826) | 0.019 | 2.410 (1.321, 4.394) | 0.004 |
| **N stage** |  |  |  |  |
| N0 | 1 [Reference] |  | 1 [Reference] |  |
| N1 | 2.683 (1.536, 4.688) | 0.001 | 2.752 (1.384, 5.473) | 0.004 |
| N2 | 3.083 (1.742, 5.455) | <0.001 | 2.922 (1.476, 5.783) | 0.002 |

*Abbreviations*: HR, hazard ratio; CI, confidence interval; CEA, carcinoembryonic antigen; VELIPI, venous emboli and/or lymphatic invasion and/or perineural invasion; NA, not available.

## Table S4. C-index comparison of the collagen^DL^ nomogram and other prediction models.

| **Models** | **Disease-free survival** | **Overall survival** |
| --- | --- | --- |
|  | **C-index (95% CI)** | **C-index (95% CI)** |
| ***Training cohort*** | | |
| Collagen^DL^ nomogram | 0.788 (0.762, 0.813) | 0.777 (0.744, 0.810) |
| Clinicopathological model | 0.700 (0.669, 0.732) | 0.725 (0.687, 0.763) |
| TNM stage | 0.645 (0.619, 0.671) | 0.646 (0.614, 0.678) |
| Collagen^DL^ classifier | 0.699 (0.678, 0.720) | 0.678 (0.650, 0.701) |
| ***Internal validation cohort*** | | |
| Collagen^DL^ nomogram | 0.795 (0.769, 0.821) | 0.763 (0.723, 0.803) |
| Clinicopathological model | 0.732 (0.704, 0.760) | 0.721 (0.682, 0.759) |
| TNM stage | 0.656 (0.630, 0.683) | 0.657 (0.622, 0.693) |
| Collagen^DL^ classifier | 0.692 (0.668, 0.716) | 0.660 (0.624, 0.697) |
| ***External validation cohort*** | | |
| Collagen^DL^ nomogram | 0.778 (0.752, 0.805) | 0.822 (0.795, 0.850) |
| Clinicopathological model | 0.693 (0.662, 0.724) | 0.754 (0.717, 0.791) |
| TNM stage | 0.612 (0.582, 0.642) | 0.667 (0.629, 0.705) |
| Collagen^DL^ classifier | 0.697 (0.672, 0.722) | 0.692 (0.658, 0.726) |

*Abbreviation*: TNM, tumor-node-metastasis; CI, confidence interval.

## Table S5. ROC comparison of the collagen^DL^ nomogram and other prediction models at 5 years.

| **Models** | **Disease-free survival** | | | | | **Overall survival** | | | |
| --- | --- | --- | --- | --- | --- | --- | --- | --- | --- |
|  | **AUC (95% CI)** | | | | ***P*** | **AUC (95% CI)** | | | ***P*** |
| ***Training cohort*** | | | | | | | | | |
| Collagen^DL^ nomogram | 0.822 (0.760, 0.885) | Reference | | | | 0.855 (0.784, 0.925) | Reference | | |
| Clinicopathological model | 0.707 (0.631, 0.782) | <0.001 | | | | 0.780 (0.696, 0.863) | 0.001 | | |
| TNM stage | 0.648 (0.580, 0.715) | <0.001 | | | | 0.693 (0.618, 0.768) | <0.001 | | |
| Collagen^DL^ classifier | 0.749 (0.690, 0.808) | <0.001 | | | | 0.755 (0.689, 0.822) | <0.001 | | |
| ***Internal validation cohort*** | | | | | | | | | |
| Collagen^DL^ nomogram | 0.821 (0.761, 0.881) | | Reference | | | 0.806 (0.723, 0.888) | | Reference | |
| Clinicopathological model | 0.745 (0.674, 0.817) | | 0.013 | | | 0.727 (0.625, 0.829) | | 0.035 | |
| TNM stage | 0.668 (0.600, 0.736) | | <0.001 | | | 0.653 (0.559, 0.748) | | <0.001 | |
| Collagen^DL^ classifier | 0.720 (0.662, 0.779) | | <0.001 | | | 0.716 (0.638, 0.782) | | 0.004 | |
| ***External validation cohort*** | | | | | | | | | |
| Collagen^DL^ nomogram | 0.849 (0.788, 0.909) | | | Reference | | 0.908 (0.858, 0.958) | | Reference | |
| Clinicopathological model | 0.748 (0.672, 0.824) | | | 0.003 | | 0.817 (0.741, 0.894) | | 0.004 | |
| TNM stage | 0.660 (0.585, 0.734) | | | <0.001 | | 0.719 (0.631, 0.807) | | <0.001 | |
| Collagen^DL^ classifier | 0.739 (0.673, 0.805) | | | <0.001 | | 0.755 (0.679, 0.832) | | <0.001 | |

*Abbreviations*: ROC, receiver operating characteristic; AUC, area under the ROC curve; CI, confidence interval; TNM, tumor-node-metastasis.

## Table S6. Net reclassification improvement by comparing the collagen^DL^ nomogram with the clinicopathological model.

| **Models** | **Disease-free survival** | | | **Overall survival** | |
| --- | --- | --- | --- | --- | --- |
|  | **NRI (95% CI)** | | ***P*** | **NRI (95% CI)** | ***P*** |
| ***Collagen^DL^ nomogram vs. Clinicopathological model*** | | | | | |
| Training cohort | 0.466 (0.347, 0.576) | <0.001 | | 0.480 (0.350, 0.602) | <0.001 |
| Internal validation cohort | 0.446 (0.321, 0.554) | <0.001 | | 0.398 (0.193, 0.530) | 0.002 |
| External validation cohort | 0.447 (0.319, 0.568) | 0.002 | | 0.482 (0.185, 0.616) | 0.016 |

*Abbreviations*: NRI, net reclassification improvement; CI, confidence interval.

## Table S7. Integrated discrimination improvement by comparing the collagen^DL^ nomogram with the clinicopathological model.

| **Models** | **Disease-free survival** | | | **Overall survival** | |
| --- | --- | --- | --- | --- | --- |
|  | **IDI (95% CI)** | | ***P*** | **IDI (95% CI)** | ***P*** |
| ***Collagen^DL^ nomogram* vs*. Clinicopathological model*** | | | | | |
| Training cohort | 0.159 (0.081, 0.232) | <0.001 | | 0.132 (0.056, 0.215) | <0.001 |
| Internal validation cohort | 0.120 (0.051, 0.192) | <0.001 | | 0.108 (0.069, 0.147) | <0.001 |
| External validation cohort | 0.125 (0.056, 0.205) | <0.001 | | 0.095 (0.026, 0.191) | 0.004 |

*Abbreviation*: IDI, integrated discrimination improvement; CI, confidence interval.

## Table S8. Adjuvant chemotherapy interaction with the collagen^DL^ classifier for DFS and OS in patients with high-risk stage II and stage III disease.

| **Collagen^DL^ classifier** | **Adjuvant chemotherapy** | | **Disease-free survival** | | ***P* value for interaction** | **Overall survival** | | ***P* value for interaction** |
| --- | --- | --- | --- | --- | --- | --- | --- | --- |
|  | **No chemo** | **Chemo** | **HR (95% CI)** | ***P*** |  | **HR (95% CI)** | ***P*** |  |
| ***High-risk stage II (N=412)*** | | | | | | | |  |
| High-collagen^DL^ (Chemo vs. No chemo) | 94 (50.0) | 94 (50.0) | 0.345 (0.178, 0.670) | <0.001 | 0.010 | 0.399 (0.166, 0.956) | 0.032 | 0.023 |
| Low-collagen^DL^ (Chemo vs. No chemo) | 128 (57.1) | 96 (42.9) | 0.770 (0.258, 2.097) | 0.638 |  | 0.389 (0.082, 1.839) | 0.216 |  |
| ***Stage III (N=418)*** | | | | | | | |  |
| High-collagen^DL^ (Chemo vs. No chemo) | 77 (32.9) | 157 (67.1) | 0.363 (0.257, 0.515) | <0.001 | <0.001 | 0.325 (0.207, 0.511) | <0.001 | <0.001 |
| Low-collagen^DL^ (Chemo vs. No chemo) | 36 (19.6) | 148 (80.4) | 0.576 (0.203, 1.636) | 0.294 |  | 0.754 (0.209, 2.722) | 0.665 |  |

*Abbreviations*: DFS, disease-free survival; OS, overall survival; HR, hazard ratio; CI, confidence interval.
